# Genome Sequence Analysis of CsRV1: A Pathogenic Reovirus that Infects the Blue Crab *Callinectes sapidus* Across Its Trans-Hemispheric Range

**DOI:** 10.3389/fmicb.2016.00126

**Published:** 2016-02-10

**Authors:** Emily M. Flowers, Tsvetan R. Bachvaroff, Janet V. Warg, John D. Neill, Mary L. Killian, Anapaula S. Vinagre, Shanai Brown, Andréa Santos e Almeida, Eric J. Schott

**Affiliations:** ^1^Institute of Marine and Environmental Technology, University of Maryland Center for Environmental ScienceBaltimore, MD USA; ^2^University of Maryland School of MedicineBaltimore, MD USA; ^3^National Veterinary Services Laboratories, Animal and Plant Health Inspection Service, United States Department of AgricultureAmes, IA USA; ^4^National Animal Disease Center, Agricultural Research Service, United States Department of AgricultureAmes, IA USA; ^5^Departamento de Fisiologia, Instituto de Ciências Básicas da Saúde, Universidade Federal do Rio Grande do SulPorto Alegre, Brazil; ^6^Department of Biology, Morgan State UniversityBaltimore, MD USA

**Keywords:** virus taxonomy, blue crab, aquaculture, geography, Brazil, disease

## Abstract

The blue crab, *Callinectes sapidus* Rathbun, 1896, which is a commercially important trophic link in coastal ecosystems of the western Atlantic, is infected in both North and South America by *C. sapidus* Reovirus 1 (CsRV1), a double stranded RNA virus. The 12 genome segments of a North American strain of CsRV1 were sequenced using Ion Torrent technology. Putative functions could be assigned for 3 of the 13 proteins encoded in the genome, based on their similarity to proteins encoded in other reovirus genomes. Comparison of the CsRV1 RNA-dependent RNA polymerase (RdRP) sequence to genomes of other crab-infecting reoviruses shows that it is similar to the mud crab reovirus found in *Scylla serrata* and WX-2012 in *Eriocheir sinensis*, Chinese mitten crab, and supports the idea that there is a distinct “Crabreo” genus, different from *Seadornavirus* and *Cardoreovirus*, the two closest genera in the Reoviridae. A region of 98% nucleotide sequence identity between CsRV1 and the only available sequence of the P virus of *Macropipus depurator* suggests that these two viruses may be closely related. An 860 nucleotide region of the CsRV1 RdRP gene was amplified and sequenced from 15 infected crabs collected from across the geographic range of *C. sapidus*. Pairwise analysis of predicted protein sequences shows that CsRV1 strains in Brazil can be distinguished from those in North America based on conserved residues in this gene. The sequencing, annotation, and preliminary population metrics of the genome of CsRV1 should facilitate additional studies in diverse disciplines, including structure-function relationships of reovirus proteins, investigations into the evolution of the Reoviridae, and biogeographic research on the connectivity of *C. sapidus* populations across the Northern and Southern hemispheres.

## Introduction

The blue crab, *Callinectes sapidus*, plays crucial roles in the economies and ecosystems of the Atlantic coasts of North and South America (Williams, 1974). Though harvested mostly in artisanal fisheries, landings are substantial: annual harvests average over 77,000 tonnes in the USA, 8,100 tonnes in Mexico, and 11,500 tonnes in Venezuela, (NOAA National Marine Fisheries Service and Commercial Fisheries Statistics, 2015; Perry and VanderKooy, 2015; Singh-Renton and McIvor, 2015). Anecdotal data suggest that Brazil’s long coastline has the potential for large harvests, particularly in the south (Mendonça et al., 2010). Ecologically, *C. sapidus* is an adaptable euryhaline predator and scavenger that can influence and regulate benthic community structure (Arnold, 1984; Lipcius and Hines, 1986; Baird and Ulanowicz, 1989; Hines, 2007). Blue crab populations are themselves subject to control by numerous factors, including predation and disease (Johnson, 1977; Hines, 2007; Shields and Overstreet, 2007; Schott and Messick, 2010; van Montfrans et al., 2010). Blue crabs are infected by a pathogenic virus, *C. sapidus* reovirus 1 (CsRV1, also called RLV for reo-like virus), throughout the studied US range from Louisiana to Massachusetts (Johnson, 1977; Bowers et al., 2010; Rogers et al., 2014; Flowers et al., 2015). Originally described in captive crabs, CsRV1 has been reported at an average prevalence of 20% in wild populations, with peak prevalence often exceeding 50% (Johnson and Bodammer, 1975; Flowers et al., 2015). While the effects of CsRV1 on wild crab populations are still not known, experimental infections are always fatal and the virus is associated with a majority of crab deaths in soft shell crab aquaculture (Bowers et al., 2010).

CsRV1 is a non-turreted reovirus with 55–60 nm icosahedral capsids and a segmented double stranded RNA genome (Johnson, 1977). The partial sequence of the putative RNA-dependent RNA polymerase (RdRP) gene and electrophoretic analysis of the 12 double stranded RNA (dsRNA) genome segments indicate that CsRV1 is closely related to mud crab (*Scylla serrata*, Forsskål 1775) reovirus (MCRV) and a recently sequenced reovirus (WX-2012) from Chinese mitten crab, *Eriocheir sinensis* H. Milne-Edwards, 1853 (Bowers et al., 2010; Deng et al., 2012; Shen et al., 2015). These three viruses appear to not be members of the *Cardoreovirus* genus, based on the low-sequence identity of their RdRP genes to that of the only definitive member of *Cardoreovirus*, ESRV905 (Zhang et al., 2004; Attoui et al., 2011). It has been instead proposed that MCRV belongs to a new genus, tentatively termed “Crabreo” virus (Chen et al., 2011; Deng et al., 2012). The relationship of two additional crab-infecting reoviruses described in the 1990s, P virus (from *Macropipus depurator*) and W2 virus (from *Carcinus mediterraneus*) to the *Cardoreovirus* or “Crabreo” virus group is still unresolved. No RdRP sequence is available in the literature or GenBank for either virus, although the electrophoretic dsRNA pattern of both species is more similar to viruses within the “Crabreo” group (Mari and Bonami, 1988; Montanie et al., 1993). The sequence of only three regions of the CsRV1 genome has been reported (Bowers et al., 2010; Tang et al., 2011). To better characterize CsRV1, and to better resolve its taxonomic position, a complete genomic sequence is needed.

The Reoviridae is a large and diverse family, encompassing at least 15 proposed genera that infect vertebrates, invertebrates, plants, and fungi (Attoui et al., 2011). Knowledge about the diversity of reoviruses is likely to expand as new species are discovered in non-vertebrate systems, especially in marine invertebrates, which historically have not been widely studied by virologists. Genetic diversity within species of reoviruses has been widely explored in insects (e.g., Mertens et al., 1999; Attoui et al., 2005; Graham et al., 2008; Spear et al., 2012). For example, studies on genetic variation of virus ecotypes or geographical isolates have provided valuable clues to the origins and movement of agricultural pests (Stenger et al., 2010). In the disciplines of aquatic animal health and aquaculture, there is a need for more studies on the genetic variation of crustacean viruses, especially RNA viruses to develop more accurate PCR-based assays and discover the origins of aquaculture pathogens (Goral et al., 1996; Robles-Sikisaka et al., 2002; Garseth et al., 2013; Naim et al., 2014). With the exception of the *Panulirus argus* virus (PaV1) of spiny lobster (Behringer et al., 2011), there has been relatively little research reported on the genetic variability of crustacean-infecting viruses in the wild. To better understand the origin and diversity of the CsRV1 virus, which has been found in every population of blue crabs sampled, we sequenced and annotated the full genome from a North American strain and investigated the genetic diversity between CsRV1 strains from both North and South America.

## Materials and Methods

### Agarose Gel Analysis of Viral dsRNA

Virus particles were enriched from homogenized crab muscle using the differential centrifugation method of Xie et al. (2005), with a final centrifugation step in which virus particles were pelleted at 28,000 × *g* for 45 min at 24°C. Total RNA was extracted from enriched particles using Trizol, separated on a 1% agarose gel and visualized by staining with ethidium bromide.

### Genome Sequencing

Double stranded RNA from a CsRV1-infected crab (inoculated from “strain” X45) was prepared from total RNA (isolated using Trizol) according to the methods outlined in Bowers et al. (2010). Complementary DNA was produced with SuperScript II reverse transcriptase (Life Technologies) using a tagged random octamer method as described in Neill et al. (2014). Products were sequenced at the USDA APHIS National Veterinary Services Laboratories on a Torrent PGM instrument using standard chemistries (Life Technologies). Sequences of genome segment termini were completed or verified by 5′ and 3′ RACE (Takara/Clonetech SMARTer RACE), and sequenced using dideoxy methods with Big Dye version 3.1 reagents and analyzed on the ABI Prism 3130xl Genetic Analyzer (ThermoFisher). Areas of poor coverage and ambiguities were verified using dideoxy sequencing of PCR products amplified using primers designed from the scaffold genome sequence.

### CsRV1 Genome Assembly and Annotation

Ion Torrent reads were assembled *de novo* using default parameters of CLC Genomics Workbench (CLCBio, Qiagen). Assembly of segment 10 was assisted by using sequence of segment 10, previously determined by Bowers et al. (2010) (Genbank HM014010), as a scaffold. The nucleotide sequences of each segment and the longest open reading frames for each segment were compared to the non-redundant nucleotide database at GenBank using BLAST and tBLASTx^1^ using default search parameters.

### Phylogenetic Analysis of the CsRV1 RdRP Sequence

An RdRP amino acid alignment was constructed from sequences identified by tBLASTx hits in the non-redundant NCBI database and in searches against the conserved domain database^2^ After initial alignment with Clustal Omega (Sievers et al., 2011), the sequences were manually aligned based on pairwise BLAST alignments and published alignments (Deng et al., 2012) using Mesquite (Maddison and Maddison, 2015). The alignments were trimmed corresponding to the conserved core domains described in Deng et al. (2012). Alignment NEXUS files are available in Supplemental File S3. Phylogenetic trees were constructed using RAxML with the Jones Taylor Thornton amino acid matrix and site to site rate variation (the JTTPROTGAMMA model) with 100 bootstrap replicates (Stamatakis, 2006).

### Analysis of CsRV1-Infected Blue Crabs From the USA and Southern Brazil

Crabs from Massachusetts, New York, Maryland, Virginia, and Florida were caught in 2012 by a combination of traps and nets and stored at –20°C as described in Flowers et al. (2015). Crabs from southern Brazil were caught in baited traps on 8 March, 28 March, and 4 June 2013 in the Lagoa de Tramandaí estuary (–29.97286, –50.15337), Rio Grande do Sul state, then held for 1–14 days in aquaria. Crabs were sacrificed by chilling on ice and two walking legs removed. Legs were preserved in 95% ethanol at 4°C or frozen at –20°C. Material was shipped on dry ice to IMET and stored at –80°C upon receipt. All procedures were in accordance with IBAMA’s regulations on capture and transport of biological material. RNA was extracted using Trizol and the presence of CsRV1 genetic material was assessed using the RT-qPCR methods of Flowers et al. (2015). RNA samples with greater than 100,000 virus genome copies per milligram tissue were further analyzed by enrichment of dsRNA using CF-11 chromatography and agarose gel electrophoresis (Bowers et al., 2010) to examine the dsRNA electrophoretic pattern. CsRV1-positive crabs with over 100,000 genome copies per mg muscle tissue were also used for amplification of an 860 nt segment of the RdRP gene. Total RNA was reverse transcribed using random hexamers and AMV reverse transcriptase (Promega Corp., Madison, WI, USA), then amplified with Taq polymerase (Apex BioResearch, Genesee Scientific, San Diego, CA,USA) using primers F2321 5′-TTGTGAATGCGAACAAGAAG-3′ and R3181 5′-CAAGTTATATTTCCATTTCCT-3. For this amplification, conditions were 30 cycles of 95°C 30 s, 55°C 30 s, and 72°C for 40 s. Primer numbering refers to nucleotide positions in the RdRP sequence included in Supplemental File S1 and Genbank entry KU311708. Amplicons were sequenced using Big Dye methods (ThermoFisher, Waltham, MA, USA) at the IMET Bioanalytical Services Laboratory. RdRP sequences from all crabs were aligned and pairwise comparisons were conducted using CLC Workbench (Qiagen). Nucleotide and predicted protein sequences are available as Supplemental Files S4 and S5. Phylogenetic trees were constructed using parsimony with PAUP^∗^4b10 (Swofford, 2003).

## Results

### The Genome of CsRV1

The CsRV1 genome was found to be 23,913 nucleotides long and assembled into 12 contigs of 1130–4300 nt (**Table 1**). The CsRV1 sequence is included as Supplemental File S1 with this publication, and is deposited in GenBank under accession numbers KU311708 to KU311719. The longest open reading frame encoded by each CsRV1 segment was subjected to BLAST analysis to identify homologs in the GenBank database. As listed in **Table 2**, each of the 12 ORFs was 60–87% similar to the corresponding ORFs encoded in Mud Crab reovirus (MCRV, Deng et al., 2012) and 63–87% identical to the ORFs of the reovirus genome (WX-2012) recently described from *Eriocheir sinensis* (Shen et al., 2015). Also identical to the MCRV genome, the 5′ ends of CsRV1 segments all begin with the consensus AUAAAYWHH, and the 3′ ends terminated with HGAWCAACKAU (ambiguity codes see **Table 1**). The CsRV1 genome lacks extensive identity with other virus sequences in Genbank; however, the 3′ end of segment 4 has 98% identity to a 650 nucleotide sequence of P virus, a pathogenic reovirus of the Mediterranean swimming crab, *Liocarcinus depurator* that is reported in Walton et al. (1999). This 650 nucleotide sequence shares 75 and 76% nucleotide identity with the corresponding regions of segment 4 of MCRV and WX-2012, respectively, and includes the conserved motif [AGAUCAACGAU] at the 3′ terminus. A Clustal alignment of the four sequences is included as a Supplemental File S2.

**Table 1:** Annotation of the CsRV1 genome.

CsRV1 Segment	Length (nt)	5′ end	3′ end	Major ORF coordinates	Protein product name	Genbank entry #
1	4300	AUAAAUUUU	CGAUCAACGAU	29–4267	VP1	KU311708
2	2720	AUAAAUUAA	UGAUCAACGAU	306–2660	VP2	KU311709
3	2706	AUAAAUAUC	UGAUCAACGAU	371–2587	VP3	KU311710
4	2449	AUAAAUUAC	AGAUCAACGAU	36–1346 1719–2378	VP4A VP4B	KU311711
5	2153	AUAAAUUAC	AGAUCAACGAU	35–1855	VP5	KU311712
6	2020	AUAAAUUUC	AGAACAACGAU	56–1675	VP6	KU311713
7	1534	AUAAAUUUC	UGAUCAACGAU	86–1345	VP7	KU311714
8	1281	AUAAAUUCA	AGAACAACGAU	99–992	VP8	KU311715
9	1243	AUAAAUAUC	AGGUAAACUAU	75–1046	VP9	KU311716
10	1211	AUUAAAUUA	AGAUCAACGAU	25–1065	VP10	KU311717
11	1166	AUAAACUAC	UGAUCAACGAU	38–649	VP11	KU311718
12	1130	AUAAAUUAC	AGAUCAACGAU	187–1008	VP12	KU311719
Summary	23913	AUAAAYWHH^a^	HGAWCAACKAU^a^			

^a^*Ambiguity code: H = A/C/U; W = A/U; K = G/U.*

**Table 2:** Analysis of predicted CsRV1 proteins.

Protein name	MW (da)	Iso-electric point	Alignment with MCRV			Alignment with WX-2012			Proposed function^a^
			Alignment length (aa)^a^	% aa identity mud crab reovirus (MCRV)	MCRV protein, GenBank #	Alignment length (aa)^a^	% aa identity recently sequenced reovirus (WX-2012)	WX-2012 segment, GenBank #	
VP1	160311	8.00	1377	87	1, AEQ75466	1368	86	1, AKC01920	RdRP
VP2	90597	6.36	785	78	2, AEQ75467	785	82	2, AKC01921	Guanylyl- transferase
VP3	84271	7.69	729	85	3, AEQ75468	739	87	3, AKC01922	Unknown
VP4A	50185	8.04	437	84	4, AEQ75469	437	85	4, AKC01923	unknown
VP4B	25273	5.44	220	78	JQ287703	216	80	KP638405	unknown
VP5	67092	5.88	607	79	6, AEQ75471	607	82	6, AKC01925	unknown
VP6	61199	9.03	539	77	5, AEQ75470	539	78	5, AKC01924	unknown
VP7	46235	5.99	419	81	7, AEQ75472	412	66	7, AKC01926	unknown
VP8	33571	4.97	298	69	8, AEQ75473	298	63	8, AKC01927	Capsid
VP9	38485	5.01	349	60	9, AEQ75474	344	72	9, AKC01928	unknown
VP10	35465	6.16	323	77	10, AEQ75475	323	73	10, AKC01929	unknown
VP11	23582	9.54	203	74	11, AEQ75476	202	63	11, AKC01930	unknown
VP12	29528	7.02	274	75	12, AEQ75477	274	76	12, AKC01931	unknown

^a^*Based on BLAST analysis.*

Based on similarity to more well-characterized reoviruses, putative functions could be assigned to three of the predicted proteins (**Table 2**). Segment 1 encodes a 160-kDa protein (VP1) with 87% identity to the MCRV RdRP protein, which is the product with the highest similarity to any other known or predicted protein in GenBank. Also based on similarity to MCRV and related reoviruses, segment 2 encodes a putative guanylytransferase (VP2, 91 kDa). Guanylytransferases are conserved among reoviridae and function to add the 7-methyl cap to viral messenger RNAs (Attoui et al., 2002). VP2 contains an Arg–Gly–Leu (RGD) motif that is shared by the corresponding proteins of MCRV and ESRV, as well as by RdRP proteins in the Banna virus and Kadipiro virus, which are in the *Seadornavirus* genus (Attoui et al., 2000). The RGD motif is recognized by cell surface integrins, and plays a role in reovirus internalization in mammals (Guerrero et al., 2000; Maginnis et al., 2006). CsRV1 segment 8 encodes an acidic 34 kDa protein (VP8) with similarity (28% over 51 amino acids) to a similar product of segment 7 of a Grass Carp Reovirus (GCRV, GenBank GU350744) which resides in a novel group of turreted Aquareoviridae (Wang et al., 2012). GCRV VP7 is highly variable but is generally accepted to encode an outer capsid protein (Yan et al., 2014). None of the other ORFs encoded by the CsRV1 genome have significant matches in BLAST searches to proteins in the available databases.

Segment 4 putatively encodes two proteins over 200 amino acids in length (VP4A, VP4B). VP4A is a predicted 437 aa protein of 50 kDa and pI of 8.04 encoded by nt 36 to 1346 that is 84% identical to the reported VP4 of MCRV (AFF57928, Deng et al., 2012). VP4B is a predicted 220 aa protein (25.2 kDa, pI of 5.44) that is encoded by nt 1719 to 2378. BLAST analysis also shows that this product is very similar to the product of non-annotated open reading frames encoded in the 3′ end of segment 4 of MCRV and SRV WX-2012. Both genomes predict very similar acidic proteins (220 amino acid, pI of 5.01 and 5.04, respectively) with 78 and 80% identity to the CsRV1 VP4B over 218 amino acids.

### Comparison of the CsRV1 RdRP Sequence with Reoviruses in Related Genera

**FIGURE 1:**
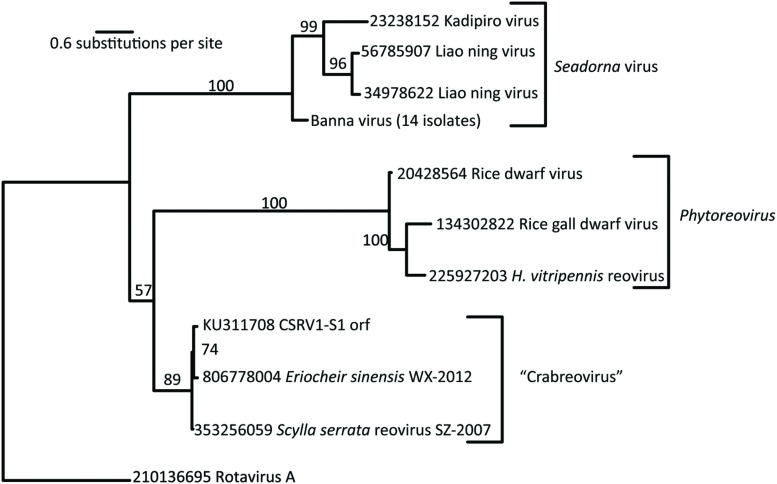
**RNA-dependent RNA polymerase (RdRP)-based phylogentic relationship of *Callinectes sapidus* Reovirus 1 (CsRV1) relative to other reovirus genera.** Phylogram was constructed using RAxML, from predicted RdRP protein sequences from the GenBank accessions indicated in the figure. Bootstrap support with 100 replicates is shown above the branches. Numbers preceding virus names are GenInfo entry numbers in GenBank, except for CsRV1, which is the Accession number. Note that the branch between the “Crabreo” group and *Phytoreovirus* is not well supported. The short sequence available from the single example of the *Cardoreovirus* group (EsRV905), did not provide meaningful alignments with other sequences, so this genus could not be represented in the phylogram.

The putative RdRP of CsRV1 (VP1) aligned well with the RdRP proteins encoded in the genomes of MCRV and ESRV WX-2012 (87% and 86% identity over the entire length), but the similarity to the RdRP of other reoviruses is below 20%, and comparisons with default parameters were not successful in aligning VP1 to any virus proteins or translated virus nucleotide sequences in GenBank. However, by anchoring alignments on conserved motifs within the RdRP protein (Deng et al., 2012), it was possible to construct pairwise comparisons between the CsRV1 RdRP and RdRP sequences of additional 12-segmented arthropod-infecting reoviruses (**Figure 1**; alignment in NEXUS format is available in Supplemental File S3). The RdRP phylogeny including CsRV1 and nine other viruses showed a well-supported close relationship between CsRV1, MCRV, and WX-2012 forming a potential “Crabreo” virus clade. Similarly *Seadornavirus* and *Phytoreovirus* also formed well-supported clades with relatively short branch lengths within each clade. The genus *Seadornavirus* was the most similar in BLAST alignments with the “Crabreo” clade, but the phylogeny suggests a closer relationship between the “Crabreo” clade and *Phytoreovirus*, albeit with very low bootstrap support (57%). Similarly, a phylogeny constructed using a neighbor-joining algorithm produced the same three well supported generic groups, but produced poorly supported bootstrap relationships between them (59 and 71%). Overall, the relationships between *Seadornavirus*, *Phytoreovirus*, *Rotavirus*, and the “Crabreo” virus clades were ambiguous. There was too little similarity between the RdRP protein sequences of CsRV1 and ESRV905 to construct a meaningful alignment, and there is no RdRP sequence data available for the other two members of the *Cardoreovirus* genus (P and W2 viruses). Therefore, the relationship between CsRV1 and *Cardoreovirus* could not be assessed.

### Geographic Variation of RdRP Genotypes

**FIGURE 2:**
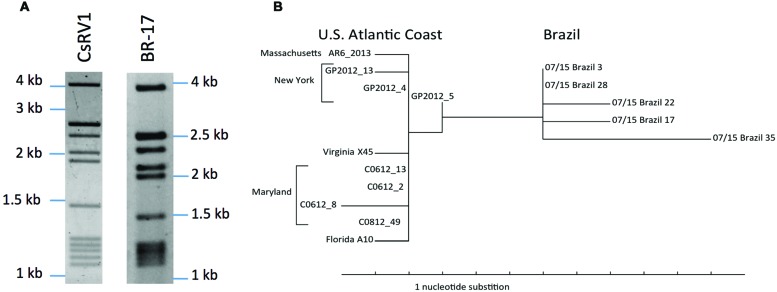
**Geographic variation of RdRP sequences. (A)** Electropherograms of dsRNA from a north American isolate of CsRV1 and dsRNA of a moribund CsRV1-positive Brazilian *C. sapidus* (Br-17). **(B)** Phylogram of the RdRP gene amplified from 15 strains of CsRV1 from the US and Brazil. The parsimony tree was produced with PAUP^∗^4b10.

Double stranded RNA isolated from CsRV1-infected crabs from Rio Grande do Sul, Brazil had a electrophoretic pattern similar to that of CsRV1 from North American strains. The gels depicted in **Figure 2A** show a thick/intense band of approximately 2.7 kb that is the result of two nearly co-migrating bands. This results in a (1/4/1/5) pattern that we interpret to be a (1/5/1/5). A phylogram constructed from the 15 aligned RdRP sequences (**Figure 2B**) clearly separates the 5 Brazilian strains from the 10 USA strains. Genotype BR-17 is distinct in that it resides on a longer branch than the other Brazilian genotypes. From the 860 nt region of the RdRP gene amplified from CsRV1-infected crabs the USA and Brazil, 797 nucleotides were aligned after trimming (Supplemental File S4). In pairwise nucleotide comparisons the 10 samples from the USA differed from one another by 0.2–1.4%, and the 5 isolates from Brazil differed by 0.3–1.2% from each other. In pairwise comparisons between the US and Brazilian strains, however, there was 1.4% to 2.5% difference in nucleotide sequence. Multiple pairwise alignments of protein translations of the sequenced region showed that no two USA CsRV1 strains differed from one another by more than 3 amino acids (3/266 = 1.1%), while between the 5 Brazil strains the largest difference was 7 amino acids (2.6%), and between USA and Brazil strains, there were as many as 11 amino acid changes (4.1%). Four of these amino acid changes were consistently found in Brazilian but not USA isolates of CsRV1; two of them were conservative (Ile→Leu, Ser→Thr), and two exchanged Ala for Thr (translations and alignments are submitted as Supplemental Files S5 and S6).

## Discussion

### Taxonomic Affiliation of CsRV1

The genome of CsRV1 assembled into 12 contigs, which correspond qualitatively to the sizes of dsRNA genome segments as reported previously (Bowers et al., 2010) and confirmed in the current study. In having 12 genome segments the CsRV1 genome is similar to other arthropod-infecting reoviruses within the following genera: *Cardoreovirus, Seadornavirus, Phytoreovirus*, and a potential new genus, “Crabreo” virus. The latter genus was proposed by Chen et al. (2011) and Deng et al. (2012), based on the analysis of the genome of MCRV, which infects *S. serrata*. CsRV1 has extensive sequence identity with MCRV, including conservation of the termini of each segment. These characteristics lend support to the inclusion of CsRV1 in the possible “Crabreo” virus genus. CsRV1 and MCRV also have genome-wide similarity to the recently reported genome sequence of a reovirus the crab *E. sinensis* (isolate WX-2012; Shen et al., 2015). While the members of this proposed genus are closely related in the RdRp-based phylogeny, the affinity of this group with the *Seadornavirus* or *Phytoreovirus* group remains ambiguous, largely because of relatively low identity in the alignment combined with the paucity of sequence data for relevant taxa with which to construct alignments. Interestingly, a cryo-electron microscopic study of a number of reoviruses by Huang et al. (2012) showed that there are structural similarities between the MCRV virion and viruses within *Phytoreovirus*. The relationship of CsRV1 to *Cardoreovirus* could not be established because of the poor alignment with the only available RdRP sequence from that genus, ESRV905. Another indication that CsRV1 is not within the *Cardoreovirus* genus comes from the differences in electrophoretic patterns of the CsRV1 and EsRV905 genomes.

Another member of the Crabreo genus may be the P virus of *L. depurator* based on the high sequence identity between a 650 nt region of the P virus genome and segment 4 of CsRV1, MCRV, and WX-2012 and the similar electrophoretic patterns of MCRV, CsRV1, and P virus dsRNA (Walton et al., 1999; Weng et al., 2007). The high % identity between P virus and CsRV1 suggests that these two viruses are the same species or may be recent variants of the same progenitor. Determining and reporting the complete genome sequence of P virus would be a significant step in understanding the relationship between these two viruses. P virus and the W2 virus (infecting *C. mediterraneus*) have been suggested to be congenerics (Montanie et al., 1993), based on morphology and genome dsRNA electrophoretic pattern. This potentially expands the number of species in a “Crabreo” genus to (CsRV, MCRV, WX-2012, P, and W2). However, no sequence data is available for W2 and its dsRNA pattern is somewhat different from P virus (Montanie et al., 1993), underscoring the value of also obtaining a genome sequence for W2.

### Proteins Encoded by the CsRV1 Genome

Similarity searches strongly indicate that CsRV1 VP1 is a putative RdRP, and VP2 is a guanylyltransferase that could function as the mRNA capping enzyme. CsRV1 VP8 is a 35-kDa acidic protein (CsRV1 VP8) that has similarity to a proposed acidic 34 kDa outer capsid protein of GCRV (Yan et al., 2014). Deng et al. (2012) also used homology searches to make tentative assignments for putative proteins of MCRV. In that study, MCRV VP8 (69% identity with CsRV1 VP8) was found to have similarity to a 130-kDa major core protein encoded on segment three of Rice Ragged Stunt Virus. Clearly, experimental work is needed to investigate which of these alternative possibilities is more accurate. Database similarity searches were not able to identify candidate identities or functions for any of the other proteins encoded in the CsRV1 genome. However, with the genome sequence now known, it is reasonable to hypothesize that most of 13 proteins listed in **Table 2** will eventually be found to represent a familiar suite of structural and nonstructural proteins typically encoded in reovirus genomes (Attoui et al., 2002; Mohd Jaafar et al., 2008).

### CsRV1 Genome Variation and the Geographic Range of Blue Crab

It is likely that CsRV1 is ubiquitous in blue crabs throughout their range. This is supported by the discovery of CsRV1 in Brazilian blue crabs, together with previously published reports that CsRV1 is present in all the North American populations sampled (Bowers et al., 2010; Rogers et al., 2014; Flowers et al., 2015). The genetic divergence of the RdRP sequence of CsRV1 from Brazil, relative to North American strains, suggests that the virus has been a part of the blue crab ecology for a long time, and that the virus genome may be a useful marker to understand blue crab population connectivity over large spatial and temporal scales. This could complement efforts to find blue crab (host) genetic markers that distinguish between blue crab populations (McMillen-Jackson and Bert, 2004; Yednock and Neigel, 2014), and to better understand the oceanic connectivity between populations of blue crab. Future geographic studies of CsRV1 genetic variation would be most valuable if they analyzed the entire genome sequence. Such studies may reveal which parts of the CsRV1 genome are under more stringent selection or even which parts of the genome may vary with latitude or variation in host life history. Investigations of the genetic diversity of mammalian reoviruses have revealed much about their biogeography and evolution (e.g., Coetzee et al., 2012), however there is little reported on intraspecific genetic variation of marine viruses.

### Genome-Based Pathogen Research and Global Fisheries

Technology that makes the sequencing of pathogen genomes faster and less expensive has contributed to an increasing awareness of the potential for disease to limit the productivity of crustacean fisheries on both a local and global scale (Stentiford, 2011; Turner, 2013). It has been possible to document and study the effects of diseases that produce visible changes in the appearance of diseased animals, such as the cloudy hemolymph of *Hematodinium* spp. infections (Stentiford and Shields, 2005). Attention has also been focused on pathogens that cause mass mortalities of crustaceans in aquaculture, as epitomized by white spot syndrome virus (WSSV), which has devastated shrimp aquaculture globally, and has been reported at high prevalence in wild crustaceans (Meng et al., 2009). Less well understood are pathogens that may kill non-cultured species and do not produce obvious signs of infection. Over 30 years after its first description, development of molecular tools to detect and quantify the CsRV1 virus revealed that it is present in an average of 20% of blue crabs on the US Atlantic coast (Flowers et al., 2015). The CsRV1 genome sequence reported here opens the door to a more thorough understanding of this virus, including studies on its origins, evolution, and virulence. Genome data will also enable the production of recombinant proteins and specific anti-virus antibodies for in-depth studies of the virus within the host.

## Author Contribuions

ES, corresponding author, had the major role in manuscript writing and editing, and coordinating data for the report. He mentored graduate student Flowers and undergraduate intern Almeida. EF conducted genome assembly, confirmatory sequencing, and manuscript planning and editing. TB conducted bioinformatic analyses. JN, JW, and MK conducted Ion Torrent sequencing, quality verifications and preliminary assembly. AV acquired, housed, and ensured transport of Brazilian crabs for analysis at UMCES. SB and AA conducted biogeographic analyses of CsRV1 genotype variation. All authors reviewed, edited, or approved the manuscript.

## Conflict of Interest Statement

The authors declare that theresearch was conducted in the absence of any commercial or financial relationships that could be construed as a potential conflict of interest.

## Footnotes

1 http://blast.ncbi.nlm.nih.gov

2 www.ncbi.nlm.nih.gov/cdd/.
