# Supplementary material for: Genome Sequence Analysis of CsRV1: A Pathogenic Reovirus that Infects the Blue Crab Callinectes sapidus Across Its Trans-Hemispheric Range
Source: Front Microbiol. 2016 Feb 10;7:126. doi: 10.3389/fmicb.2016.00126 (PMC4748042; doi:10.3389/fmicb.2016.00126)
Supplement: Supplementary file 1 [file Data_Sheet_1.PDF]

Supplemental files for:

Genome sequence analysis of CsRV1, a pathogenic reovirus that infects the blue crab *Callinectes sapidus* across its trans-hemispheric range

Emily M. Flowers[1,2], Tsvetan R. Bachvaroff[1] , Janet V. Warg[3], John D. Neill[4], Mary Lea Killian[3], Anapaula Sommer Vinagre[5], Shanai Brown[6], Andréa Almeida[1], Eric J. Schott[1*]

[1]Institute of Marine and Environmental Technology, University of Maryland Center for Environmental Science, Baltimore, MD, USA;
[2]University of Maryland School of Medicine, Baltimore, MD, USA;
[3]National Veterinary Services Laboratories, VS, APHIS, USDA, Ames, IA, USA;
[4]National Animal Disease Center, ARS, USDA, Ames, IA, USA;
[5]Departamento de Fisiologia, Instituto de Ciências Básicas da Saúde, Universidade Federal do Rio Grande do Sul, Porto Alegre, Brasil
[6]Department of Biology, Morgan State University, Baltimore, MD, USA

Running title: Genome analysis of a blue crab reovirus
Keywords: reovirus taxonomy, blue crab, aquaculture, geography, Brazil

***Correspondence**
E. J. Schott
Institute of Marine and Environmental Technology
University of Maryland Center for Environmental Science
Baltimore, MD, 21210 USA
schott@umces.edu

Supplementary files.

S1. CsRV1 genome sequence.

>csrv1-S1 4300 nt

ATAAATTTTAGTACACTGGATGCGCATAATGGCTGAACGGTTGAAAGAACTACAGAGAGAAATTAAAAGAAAGGAGAAAGAAAAGACAC
CAACCCCCAAGGATGATGAGATTGTGGCTCCTATCCCTACTGTGGATGAACCATCGATCCCGACTAAGACGAATCCTTTTCTCGATTCA
ACCACTTTTTCCACACTACATAACTCATTGTATGGCAAGCAGATATCTAGTGTAGATGAAGAGCTAGCTAAATTGTGTGAATCAGAATA
TAAATTACAGCAAAGGATATCTGAAGAACAGATCAGTGCCTTAAGACAATTTCTAACTATTAGGAGCGGCGCACCTCAAGAAGTACAAT
ATGTGGATAGAAACTGGATGGAAGCCAACAAACACGTTTCATCTTTCCTGGGAGATGTAAAATTGATGTTTGGAGACACAGCAGGCAAG
TTTCGTTCTACTAGTAAATCTGTGGACTCCATCCAGAGTGTGACATCAGATGTGCAAGTAACACAGAGGAAGCAGACACGATCTGAAAT
ACGTAACTCTTACCGCGTACAGAGAAAAACCAAGGTTGAGCAACCCCTGAAACCAAACACATTGTATGTGTATACTTACAAAGGATTAG
CACGCGTTGTGTTGCGCTTCGTACCGAAAGTAGAAACTTTGCCTGATGATGCGAAACAGACTGGACAGAAGACTACAGCATTCACATAT
GATGATTTGAGCCCTACGTGGAAACATGTACTCACAGAAGCTAAACGGGCTTTTCCTAATCATAACTATCCCGATTGCATTCATCCCAT
GACATGGTCCCAGTGGTTAGAAGAGAATAAAGATGATGTGAAAGTGTTAACACAGTACGCACATAAACTTGACTATGTGACCCTGTTGC
AAGATTTCAATTTATATGTTGCAAAGGGAGCGAGTAGAGTACGTAATATCGATCTCTCAACTTTACCGCCTTCCATTAATGTCATGGAT
CATTTCGACTTATATGGAGACGATAGCATGAGAGAATACATCAGATCGGGTGAATGGTATAATCTACTAACACACGCAGAGCAGGAAGG
AGTAGAAGTGAATGATAGCGAGAAGGTCTTTGCCAATCCCGATACGTATGTGTTAAACCTTAAGAAGTACTTTCTAAGAAGGTTTCAAC
AGGAAGTTGCTTCCACTGGGATGACACCACTGACTGATGAACTGCTCAATATCATGTTTGTGCACTGGGACATCATTGTGAATGCAGAA
CCTAAACTACAGGTTATTAAGGATGATCTCCTCAAATACTACTCTAAGTATGGTGTGGATGCCACGTTTGATTACAATATGAAGAGAGG
AGAAATGGCAGTGGTGACGCGTGGACATTTACTGGCTCATAAAGTGTTAGAATGCGCACTACGAGTTGTTGAAACGATCTACACGTACG
ACTTGAAGGATGAGAAGTTCAAAGATATCTTAATCGACTTGGGACGATTGATTATGAGAGATCCAATATATGGCACAACTACTGTACGT
GACGCTACTACTGTAATGAAACAGCTAATGTACACTCAGGGGAAAGAGTTTAGAAGAATTATATTTAAGAAGTATGATTACCTCAACTT
CAATGAGCCTCTAGTATTAAAAGGCGATGAGCTGACTGATCAACCTCCTACACTACTAGCTACCACTCACTATGAAGAAATGGATAAGA
AAAGGATTGACTCTTTAATCAAAGCGAATGAACACGCAGGTAGAATCTTACCTATGTCAAGCATCTACCGTTGCAGATATACTGATTCT
CTGGACCTCATCGGAGACGCAAATAGATACTTCTCAGCTTTAACGACATTGGAAGCAGTAGCAGGATTCGCTAGTTCAGATCTTTTATC
CGGATTTATTGATAGTAATGAGTCAATTGAATTCACTGGAACTGAACGCCTACGTAAACTGCTGTATTATAGCGTAAGAGAGCAAATCA
TCACCCTCAACACGTCAACTGTGCCAAGACCCTCACTGCTAAAGGTGTTGCTATCATCTGCGAAAGACACTGCCTCTGCATCAGTACAA
CCCATCACATTTAGAATCTATAAAACCACACCTGAGTATGATGGCGACACCTTGAATATGGTGGAGTCAACAGTGGAAATGTCAACGCG
CCAAAAGAAGCCAAATCTATTGAAAGCCGCGGAAATACTAAGATCTACAGTCACCACAAATGCAGAGATAGTCATCTCAGGCGGTACAC
GTGCTGTGCAAGGTGGGAAGGGAGCGAGAGCCGTGTATCCTACTAAACAGCCTTACCACATAGGAGGATCCTTGTTATTTCACAAGGTA
GATGCTGTTGTGAATGCGAACAAGAAGTACCGTAGTGTTTCCAACAAGTATGGTCAGGGCATAAGTAATGCGATTCCACATATAGGTGT
ACCCGAAGTGATAGCAGTGTCTTCGGATGGACTGGCTGTTTGCTTGGCGTTAGATGTATCTGCCTTTGATGTCGCTCAGAAGTATACTG
AGACAGAAATAGAACTTGCGATGCGTGATGGGTTCCTTGACTCTGAGACATCTATGGTTTCTGGAGAAACGGTGCTTGAGCGTATGAAT
CCAGCAGACTTGGCAAATAATCTACTCACAAACACACCTCCTAAATATAAATACCAAACGGCATTGGGTGATATCATTATTTTGCAACA
TGGTAATAGGTCAGGTGTACCCTGGACTGGAACTCAGAATGACTTAGTCAATGTGAGTAATCACCATATGGCTTACGATGAGTACAAAA

AGCGTGTTGCGGAATTACAGCGACAAGGAAGAATCTCTGTCAATGTTAATGACAAGCATCACATCGTTCGTGTGTTTGGAGATGATTCT
ACATTCATCATGACGTATGATGAGCCACCTACTGCTGAGGAAGTACATTTGATGTGTGCCACTTTTGTTGAGAGTTACCAAGAAACTGC
AGGTACACTAGGATTCGCCATCAACGCTAGGAAAGGAATGATAGGTAGATACGGAAGTGAGTACCTCAAGAATACAGCTATCTATGGAA
ACATCAAATCAGTCAATCAGGTGAAGTTCCGTGGATCTGAAAAGTCGGCAAGTTACCATTTTGGCGTTTCTGAGAAGGTGTCTATGATT
AGGGATATCACAGACTTAACCATCACTAGGGGCTGCGATGAGACAAGGAAATGGAAATATAACTTGATGATGCTACCAGTGGACCTAAC
GACGCGAGCTGGAGCGTTTAGAATGTACAATCTGTGTTCCATAATGACTGGTGTTGGTAAGATGTACCTAGGAGGGACACTCAACAATA
AACTAATTGCTTCATACCATGGGCACCCTTATGGCTGGAACTTTGATGACAACCTCATCAAAACTGCCAACTCTATCGGTGCTATATCT
GACTCATCCTACGATGCCATTTCTACCAAGATAACAAACCTGCCTGACTTTAAAGATTCACAGAGAAGGATAACAAAAGATGTAGTCAC
TGCTGGTAGGCTTCCACAACATCTCAACAGGTATGGCAAATCTAACATATTAAGGCATATATTGGCGTCTGCAGCGATGGGGCCACTGT
CACAGATTGAGAAGAACGTCAATGCCTACAACGTCGCTATAGGAATAATGGGAGGTAAGCTGGAAGCACCGACTGTCCTCGAACGGCTG
AAAATCGGATTCAAGTATGTAGTGATGTCTGACCTTAATCAAGATGATTACTCACCTTACTCTTGTCAGGGCCTACAGTATAGAAGGAT
GTTGGTTCATTGGGGATTAAATGACTCAAGGATCACTTCCTTCGACCCAAAGAGCAAGCTGCAGCACCTATTAGCTAAGAATTCTCAGA
TCTTGCCAATACACTTTGATATCGAATTCGTCTACAGACTCTACTTGCAAGCTGGTACGATGGGATTCTTGCAGGTTATGTCATACTAC
CAACTACCTGACACACTAACTCATGAGATGCTAGCTGCTGTTGTCTCTTTGGAACTACAACTTGGCAATGATAAATACGCTGTTGATAT
GGGTGTATACTCTAGTCAGGCTGGACAGATACGTATAAATGATGCACTGATGGATAGTATTATTCAGCATAGACGCGGACCAGCATTGC
CCATTATTGATAAGACCTTAAACCGTCTCCTCCTACACACATACATGCTTATGTTTGGACTGATGGGGAAGTCGATCGATAGTACTAAA
GTAGACCCTACACTGAGTTGGCGAGcCATATTGGAATCCAATGATCAACGTGTTGCGCAACTCTCTGAGCTTCTCACCGCCGTGTAGTC
GTCTTGCGTTGTACTAACGATCAACGAT

>csrv1-S2 2720 nt
ATAAATTAAAGCTACAGAAAGTGTCCCGCTCATACTGGCTGAATGACTCTTCTCAAATCGCGCCTACATTCTCTAGATGTGCCCACTGC
TAAATTTGTCAAGCATGAGGAACTTGATGAGCAGATTCAGATTCGTAGAAGACGGGGGATTCTTCCTATCGTTAGTACGGGATCGAATG
CTAATCAAATAATCATGCGACACGCCTTCGTGTTCTCATACCCAGGGCAAGAGATACTAACAACCTCACCAATGCTCGCTCATCTGATA
AATAAAACAAAGAAAGTATCTCGAGCCAGATCGGCGATATGTTGAGGGAAATATTTATGAGAGATGGCACGATTCAGATTGGTTCAGAG
TTCAGACAAGCCGAATATAAAGTGAGAGAGGATATAGACTGTCAGTTGTATACCATGGAAGAAGATTTCATGATAGACGATTTGATTGT
TGGAGAGCAGATCAGACTGAAGTACCCTATAGTGGATGTGCCTAGTGATACACTCAGAGATGAAACTGTTGAAGATTTTGTCCATGTTA
AGCAAGACAAGGTACTGGGATTGGCTCGAATTCGGAACATCACACTAAAGGGTGGAAAGGAAGCAGTACTGAATAATCTGAAAGAACCA
AACCTATTCAGAATATCGAGCAGACAAATCGATGAGTCGATCATCAGTAGTTCTAGTCCATACGATAGGCCGATTCAACTATATCGTGG
TGGTCAACTTAAACTACTCTGGAGCACACAACCCGAGATACTTCACGCTCAGCCTAACTTGATCATATATGTAGGTGGAGCACCAGGTG
ATTGGGTTAACCATTATGCTAAGGCTAACGCTAAAGTTAAATGGATTTGTGTTGACCGTCAGATCCCTAAGTATCCATGTCAGCATGTG
AACGACTATGTTACACTAGACAACATCCCTGCACTTCTATCTAAGATTGATGATTCAAATCGAGTCATGGTCATCTGGGATGTGAGAAA
GTTGAGACCTGTGGGGATGAGCCGAGAGCAATGGAACGAAGTTGTTTCTGGTGAGTACGATCTTGCGAAGGCCTTTCTCGATGCCTGTA
TCGTCAAATTTGGTAAAGTATTCTGTCATGTAAAACTCAGACCAGAATATCAAAAGCGCCAGACTAGATACATTGCAGGTACCAGCATT
AAACTCCAAGCTTTCAACAGGTTAGATTCTCATGAGACGAGATGTGTCGGTTGGATAAGTGAGGTCAAGGATGCACTAATATCTACGGA
GAAATACATCGAGTTAGTTGATCATACTTACAATAGGAGAAGAGATCTAAATTACTCATTAGATCTGAGAGTCATTTCTATGAGGCTGC

AGGAGGCAGTTTCGAAGAAAGTCTCAGCGAATGAATTTCTCCAAGTGCCAAGTGATCAGATGGTAGCATTGTTCAGTCTGTCAAATGAG
ATTAATCTGAGTAATAAAGAAGAGATTTTTAAGAAAATATCTCAAGGATCAGTTGTAACGTTGGAGTATGGAGGACTAGAACGGCAGGG
TGAGACCTTTGGTACGATTGTTGATGGGAGAGAATATCGTGATTTCTCAGTAGATATATTGGATGACGTCACTCGGCATGACTGTACAC
TACAACCATTATGGCATATGTTTGCTGTTTCTCAGATGGATTATGTCGGGGACTCTTTGTACAGTGTAGTTTTAGCCACCCGCCCGAGA
CATACGTACGAACAAGAGACGTCTGTCACTACGGAGATGGTGAAAGCCGTGTCAAATCACCTTAAACTCAACTACTTTCCGGATGACCA
AGACAGAGTATACACTGTTAGAAAAGAGGTCATTGATGTATACTCTAAGGAATATGGGATTGTTGGTATTGGCTGGAGAGGAGATTACA
GACTGTTGGATGACAAATTTAAGGAAAAGAGGTCTGTTTCCGGTCATCTACTATACGTGCTTGTCGGTGCATGCCTTTATCCTATGGGA
GTGCGTAAGTATGTACAGGTGGTAATCAATAACTCACAAAACATGTCCATAGGAACTGAGTTGAAAAATCTGACTGAGGAAAGAAACAG
GTGGCACTACATATTGGACTACATTCTTGCTACGTACGCTGCAGAGAAATTACTGGCTGAAATGGTATCAGGATTGACCAACCCTTGGA
ACGTTGATAGATGTTATCGAGCTATTAAAATAGTAAGAGAGCAGCTTGAGACGTACTTAGAGTGTCCTTCTAGCGTACCTGTCATCCTC
ATTCCTGCGATGGGAGGGAAGTCCAGTCTGTCAAGTAAATTTGACCATTTGCACGACATAGACATATGGTACGAAAAGACTGGATTCTT
TGCAAAGAGGGAGGCAGGTATGACTGAGCAGGAACAAGAGGACGCTTACAACGTTGTACTTGATACCATCCTGCTGGATTGGCGATCGA
TGAGAGCGAAAGATGTTTTACTATGCCATACTGTGTCGCAAGCAGAGCACCTAAAGGCCAGAGTCCTTGGAGGTTTTATCCCTAGTAAG
AGATTGCAGCGCATCGTCATACAAAAGGAGAAACCAATTCCATTGAGATTAATGGTATCGAAGATGAACATACGAGATATTTCGGAAGT
ACCTCATACTGTCTACGACTCTTTCTCAAGATTAGAAGAACTTGTCATTGAAGCTGCAAGGCAGGCCATATGCAAGAGTTAGAGCGTTC
AGTGTAGCAGGCGGGTATGGTCCTTACATTCTCTGTAGTTGATCAACGAT

>csrv1-S3 2706 nt
ATAAATATCGTAAAGGAAACATGGCTAGTACAACTAGACTTGTGAATGACCGAAAGCAGTTAGAAGAAGAGGTTAAAGAAGATGCTCGA
ATTCTTGCGAATGCGCGAGGACTGAACATCACATCCATCGCCAATGATTCTTCATTCGGAGGACAAGAAATTAGAAATGTGGGACCAAA
TGAACAGGCGACGATCAATGCACTTAACAATGCCATAAAACAGATTGAAGCTTGAGTGTTACAGTAAGCAAGACTGAAAGGTTAGACGA
CGCTCAGATACTCAATCCCAATGTGTACACCCAACAGCTCGAAGATATCTACTCACCAGAAGAAAATGTGTACATCGTCCTCCCACGGT
TGGCTTTCCCTAACATGAAGGGTTTAATTGACCGTAGAGATGCATCACCCACGAATTTCACCTTCTCCATCGCATCCCAACTCATGACT
CAACTGTCTGCCACTACACGTACAAAGATTTTCACTGATTACTCAAAAATTGCTGCCAGCGCACTAGGGCCTGAGATCTCCACTGAAGG
TATACCATTAGTGAGTCTGATTGATAAGTTTGGACTGACTGACGCGGAAACGTCAAGGCTGCCCGTTATCCAGGATTCCATGGTGATAC
AGAAAGGTGAGGTAACAGTCGGGAATGCAGAGCAAGGAGTCAGCACTATCAATATTAAAAGAGTACCCTTCGTAGGCTCGGGATTTCAA
CAGATGATGGATGATCTCCTTTGGAAATATAGCACTAAGTCACTCACCACCAAAGAACAAAGGAGACAGAGGATTGTAGAAATGGTAAA
TGACAGGAGAATTATGATACAGAATCTGACAATGGCGGAAAAACCACAAGTGATGAGGCATGTGTCCACAGAGATTAATAACGGATTAT
TCTTGAAAATGTCGCCAGTTGCTCAACTCTACATCTACCATTTAAACAGAGCTTTTCTTGACGGCGTAGGATTCACTACACTTGCCGAG
AAACAACAGCAACTGCAATTGCAGCTCAAGACCAACATCCTAACTGCCAATCTTATCAGATCAGCCATCAACGGCATGAACACTGAGTC
GAACATGGAGATAGCAATTAAAATGATGCAGGCAGCTCAATTGCGTAAGGCTCCCATCGAGATCGCCTTTCCTATGAACGTGTCACTGA
GCCCCGAGATCATCGTACAATGTTTCATCATCTGGATGTCAATCCCTGAACAACTGTTAAGTAATAGGTCAAATTTCACCATCGCTGCA
GTAATCTGGGCAGGATTTTCGACCGAAGATTCCTACGCTGATATCATGAGAAGGAGCGCTCGCGCTTCAGACAGACAGAACTATGACAT
CATCAAGGCTGCACTTTCTAGTAGAAGATTCAAACTGCCCCGAGCTTCTACTACCCTAGTTGATGAGAATGAACCTGTGGTACGTCGCT
ATCAGATAGGAAGAGTGTACGCGCCTTTCAACGTTGATCGGTACGGCAGTCCAGTGTACAGCAATTGCACAAAGGTAGAACTTGCTGCG

GACTACAACGCAGAAGGATTCACAATCAGGAAGGATGATTTCAGAGCGTTGCAAGCTATACTTAGGATTGACGAGGATAGAGCGGCTGA
CATGTTCTCTACCTTGCGTGTGATGATTTCATCTATCCCCTCAGTCTGGTATGATGCTGATGTGGTTCACTACCCCCATGTGACTGTAG
AACTTGAACCCTTAGCAGCATATGGACTTACTGGAGCGTACCCTAGAACCAATCATAACGTAAGTACCATCGTCAAGACCATCAACAAC
ATCAGTGCCACATACTGCACTATTGCTCAGATGCTTTCCACCATTGATTTGGATCCCTCGCGCTATGGTACCACTGAATCCATAGAGAG
ATTCAAGATAGGATGGGAGAACGTCGAATCGATCCTTGAGATGGAGGGCAACGATTTTGTTAAGACCATTCTGTATGCCTATGAGGACA
ACTTCCCCAAGAAGGACTTCTACATGATGTTGAAGCAGATCGCTAGTGATGGTCAGGGTGCCCATCCAATTGCAGCCGCGATCGATGAG
TTAAGTACTATCGTGTATAGACAGCCAGAGAGGTTTGGGTATATAGACTCAGTCATTCTTACTCATATGCCTGACGTTGACACTGGATA
TGATAGGTTCTTCCACCTTCATCCTATTGTCTCCAATCAACCATCAAACACCATCAGAAATGCTAAGCTGTGGAATGATATGAGGTTGG
AACTGCAATTGGAGTATCTGAAGGCTGGACCTGTTAGAGTAGAAGGACCATTCCATGTCACCTACAACTTTCTGTCTGAGGAGGAGACA
CTGCCTGCAACCAGTCACATAGTCATGAAGGATAACATGATGCTCAATGATCATCTCACGTTCAACTTTGTGAAGAGAGAAAGGAGGAA
CAACAGAAAGAAGATCGAGTCATTCAGGTACAGAGTGTCTGATATGTACGTTGCTGTTAGAATATCCAAATTCCAACTGGAGATTCTAC
GTGACTTGCACGATCTTGTCAAATCCCGCACATATCTGGATACTTCAAAGAGTCCACTTGCTACATCTCCCATTCGTGTGGTTGAATAT
GTGAGGTAGGGTGTGAGTGGCTTCACTTCCTATCGATCATAGATTTCTCCATTGATTTTAGTGTCGGTCTGTGTTCAATAGTTGTATTA
GCCTTCCTACCTTTCGCTAGGGTGATGATCAACGAT

>csrv1-S4 2449 nt
ATAAATTACAGTGACAGTTCATCGTCGCCCGAGAGATGTCAGATCGTTCGCGCATTCAAAAGAAGAGACACCAATCTTATCTTAAGTTA
GAAGACTACGTACAGGAACAGCACTCCAAGAACGTGTCCCCGAACTCTAAGGTGGACTGGTCTCTACCGTTGGTTTGTGATACATCAGG
AGCTGGTGAGTCATCTTGCAGAATTGAGCGAACACAAGAGATACTGAAGACAGATGAACGTGCTGTGGATCGGTTGTTTACAAATGTCA
CTAGCAAGCGGTCAAACCCTATTACCTCTCAATTCTTGGACAAAGACATAAAGCAAATCTACACCAATAAATATCAGACGTTGAGCTCA
GTCCATGTAGCTGTGGCGAAGGAAGAGATCCTGTCACTCATCTCTGAGAAGATAGGCTTACCGTCCAATGTATTGCTAAGAGTGGATAG
GACAAATATCATCATAGTTGGACAGATCACAGAAAAATGGTATCAGATAGAGTGTGATGCTTCAATTCCCTTACTTTCATTGTTTGTTC
ATTCCATTCCCCCTCTTAAGAAGCTTAACAACAATCACTACATCTCCCATTCTCCTTCCTACCTATCATTAGAAGACACCTTTCAGACT
CAGAAGATTAACTTCGCGAATGGAACAGTATTTGCAAGAAAGGTACTTAGACACACATTCATGGTCGGGACTGGAACCTTAGAACCATT
CTGGCACTACACCTTGCCCCAGACTGACGGAACGTGTCTATACTACCATGTCTTTCCCCCTGTTTTAATGCTAAATCACCATTTAAGTC
ACAAGCACTTCAAGCTATCTGTTGATATGTTCATCCAGTTGCTAGTGCGTAGAGGAGGTGCATTACATTGCGACATCAGGAGACGGAAT
ATAGCAGTAGTAGACCACAGACTTGTATTGATTGATGATCATAGACAGGACGGTGGGAATATCTCGCAACTAACCCATACTATTATGA
TTGCGGAGATCAAGAGGATTTGATTCTACCAACCACCTTCTATCAGGATCTCTTAGCTTCTTTTAGAGTGATGGCTTCAATATCTGGTT
GTGTGTATCCCTGGAGTACTGATCGTAATGCTGATGCGGTGACGTTGTTATTTGATAAAGATATGAAGCTAAGTGGAGATAAGTATCAT
ATTACTATGACTTATGGAGATATCTATCAACTAAGTGTGTTAATGCGCAAACACATGCGTAGATTAGCCAAACCTAGCATTGACATTGA
ATCTATGATACTAAAACATCTCACTACTCCTACTACATCCCAGCTTGTCGCCTTCAGGTGTCAGCAAGAGACGAGCGGCATTAGTGATC
ATGTCGACGGATGAATGCATGTGTATGTCTCTTTACTGTCTACTACTTCCTTCACACATATACTTTCTTCTTCCACAAACGCCGATATA
TCGGTAGGGTGCGATCTCCTATTGAAGAAACATGCATCTGACTCGACACCAGTTGCAGCGCATATGTAAGTCGGTAACGTCATTCGTAA
CCTGATATCTCACAGGGTAGGCACTATGGCTAGAACCCATAGCGGTAGTTTGTGCAGCGATTGTCCGGTGGCGTATGTTTGTGAGCAAA
GTAGCTTCGAATAACTCCTCACAATGTTCAGTACTAAGCCCTTCGATCGCCCCGGCTGCCGTAGAAGGAGAATCTCCGGAAACGCTATC

TCGAATGATAAGCCGCAATAATATATTATGGCAGATGACTTTTTGGAACACTTTGATACCCACTTGAAAGACTATAGTTTTACTTTTGA
CCCCAACGATCAAGCGAAGACGGAGAAGCAAATCCGAAAAGAAAGAGAGAATGTTCAGATGCTGATGTTTCAGAGAGGGATACCTCAAC
AACCTCGCGAATATTGCACTCTGGCATCTGAATCGGACCACCTGTTATACACCGTTCATTCCCTTGGAGCCAGGGCAGCTATTTGTAGC
ATACAGAACTTGTTGTACTATCCGTTGAGTGTCAATGCACCTTCCAGATTGAGAGGATTGGTGACATATTTACGGACTCTAAATTTTAG
CAATGAGACTAATGTGATAGGAACACTCCAGAACCTGAAGGATCATCCATTATTCAATGAATATATCAGTCCTAGCTGGGACAAGATTA
AAGCCTTCCTTATGATGATGAGTGCGTCAGGGAAGCCCCAGTCTGTTTCAGATGATTATGAAGATTATTTGACTGATGACCGGATAACG
ACCGTGACTTTCAAAGGATTTACCGTAGTCCACGCCATCTCTTCGGTAAGGTATGGTAACAGCGAGATGGAGGGAGAGTTCTACACAAT
TAGTGCGACCTCCGCTGATATGTTTAGACACTTGCTGGCCAACTACCACTATTCAGAGGGCGCGTGAGGGATGCAGGACGCCACACTCT
CGGTTTGAACTGAATTACTGCTGGCGCAGGCACTGAGATCAACGAT

>csrv1-S5 2149 nt
ATAAATTACAGTATCACATATCCTCAGTGAAAACATGGCTTTCAGAACTCGTCTAGCACTTCGTGGACCTGATGATGTCTTTGGCAAAG
GAGTACTCCGACGTCAAGACTATGGAACCACTGCTCAGATTAACGAAGGTTTAACGGTTTTCGACTCTGATGGCATCGAACTCGCGACG
AAGCTAGATCAGACTACTCGTAATGCCAACATTACTCGAAAAGGTTTGAATACTGCAATTGCTGACATTGACTCTGAGATTAAACATCT
TGCAGAAAATCTCTATGAGACGGAAGGTGAGCTTAGTCAGACGGAAAAGAATGTCGTTCGGACAGCCCAGATGGTTGGACACTTTGAGC
GCGCGGTTCGTCGGGACATTGCTATCATTAACCAGAACATCCTGAACAACTCTGATCAAATAAATCAAATCAAGAATGATCTAATAGCC
TTGGTTGAGAATGTTCGCAGTAACACTGTACAGGATATAGTCACAACCACTGCACAGATCGCTGAAAATATCGGACTAGCTACTGGTGT
GACTGGCGCGATGATTCCTTTCAACTTTGTTACCTCAGCTACAACTGACGCAACAACCGCTGTTACGGTCGCAACTGATACGGTATCCT
TAGCAACCTCATCTTACAATCTAGTTCAACTTCAGAAGATATTGAAACATGAGTCATCCCTCAAGGAACTAGTGCCAATATTCGCAAAG
GCAATCATCAGTGGGAAGACTGCTGCCGAAATTACTGAAATCATTTCGCGAGTTGGTGATCACAAGGTGGCTAAGGTTAGCACTACGAC
TCATGATATGGTGAAAGGAGTTGAGAGGCATCGCATGAACTTGTGGGTGGATAAGCGTAAGCTGAATCGTGGAATAACCTCTGGTATCT
TCGAACAGTTTAAAGATTATGGTATGTTTGTCATTGAAGCTACACAAGGGATATTGCGGCTTCGTGTCTATATTGATATTTCAGCTAAC
ACCAAGCCTTATGTGGTGACGGAGCTACGCAACAAAGAGGATACGGTGGATGATTTATCAAGTGATTTTATCGCGGTACCCCGCGATAC
TCTCGCTGATGTTGAAACTATGTATCATCGCTTTAGTACTCTTGGTGACTACATGAAGAAGGTAATCAATGTGGATATCTCATCCTTGC
AAAAGCAGATCGTCGCTATCATGTTGGGCGAAAAGGACGCTAGCATGGGACAGATTGCTGAGTTGACTGAGGATCTAGATAATCTAGAG
ATGCGAAACTTTACAGTGGTGGACACCTATGCTTCACATCTTGAGGCGAAGCAGGAATTTGATCATCATCCTGTGATCGGATCAGTAAT
CACCACGTTCCAAGGATTTGGTGAACACGATGAGCTCATTTATCCTCCTAAGGGTGTTCACGAATCATGGAATCTGCTGATTGCAGTGA
AGCGAGAACTCTCGGGACACGCTTACATTGGAGTTGGAGATCTTGTTCAGCGTTTGGATGGCCCTACCTCGTATACACTACGTGTGCCT
CATGGTCCTTTGCGTATAACAGCGCGAGATGTTGATGCTATCTTAACCGCCTCGCCGGATTCAGGGAAAACAATCCATTCACATCCGTA
CGCTCATCACATGAAGACGGCTGGATCCATGACAGACACTAATCCTCGATTCCAGGACACTGACTTGGGGAAGAGGGTCACTTCTTTGA
CGAAAGGGTTGGGAAGAGCTGCTGTAGAAGAGATTCTCGAGCGTCTACATCAACCTCATCCTGATAAGGGAAATCCTGACGATCGTTAT
GGAGCTGTGGATTTCTTGTCTGCTGTTGGTAAGAGGGTGTTGAAATCATACCAAGTCAGTGGCGTTGTCAGCGATTAATCCTTTTATAG
TTTAGAGTACACTCGTAATATTATCAGTCTACCTGTCTACCTCGCATATGATATACCTCTTTCACTAGTAGAGCGATAGTGATCGCGGC
ACTAGTAGAGCAACACTCAGATGGTCGATCGACTAATTATTTATGTATTTATTTATTTATTTATTCATCTATTTACTTGTCATTT

ATTCATTTCCACATGTGTTTAAGCTACCACAAGTGCGTGAGAGCGATGACTGTCATGGTGGGGCTCCGACGGAGTAGCTGAGTTGTGAT
CAAGATCAACGAT

>csrv1-S6 2020 nt
ATAAATTTCTCTGCAGGTCGATTGCCGCGATCAACGCGGATAGCACGAACTTAGAATGGATCGTAATCGAACTTTTCAGATTCCTCAAC
ATGCTCCGCACCGAGACATATTCTCTGCTACGACAGATGAGGTTGCTGAGATGCACGAGCAGCTGAAAAGCGGTTCATTACTGAGAGGT
TTTCTTGTTGTCGAGCAAGGACAGGGGCCCTATTTTGTTGTTGTGGTGGACGAGTCAGTACATCAAGGATGGATTTACAATGCACGTAC
CACGAGCCTTGAGGTGCGAAAAGTGACAGATGGATTATGTATTGGTCAAAATGTGGTACCGGACGATCAGGGCATCATGCATCCAGGAA
AAGGTGAACCATACCATGATCTGTTGATTACACTTCTGAACGCCGGTCTGATGCCTTATCATCAATACCAGCCTTCAACTGTGTCAGGA
GCCATTGATTTGACTGAGGAGTACAGTACAGAGGCGGTACAGTTAGAATCACTTGGGAAACAGACTAGCAAATTTGAATTCTTTCCGGC
TCCTAGTCTTTCAAAGTTGAGGAATGCGAACATCCCGTGCCGGCGTATCTCTAGAATTGAAACAAGTAAGTGTGATTGGCCTCACGTGT
TTGTTCTGGATCCATACAACTGTACACTCGTCCGTTTACCAGTTTTGCGCACATTTTCTGGACTCAGTTATGAAGGGGGTATTCAGCAG
GACAAGACTGTGTATCTAGTATATAATCCATTGGAATTCATTGGCATATGGTATGTTGGAAGTGGTAGAACTGTGGCTTTACAGAAGGT
TTGGGTGAAACGTATGCTGCGTGATGCTATCACATCTGACTTTTGTGATACTGTAGAGCTGCGAGGAAAGGTCACGCCTACAACCCAGG
AGCTGTTCACTGACGCTGTGAATAGTAGGTCAGGAAGGAAAATGAATAATGCAGGATGGATTGAGATGCTCTTCAAATCCAACCTCCCC
AGGTTTGTGATGTTGAAGAGGCTTCTTTCTGATGGTGACATGAGTAAGATTCAGATTCAGAATGAGGCTGCGAGGGATTACATCTTGTA
CGACGGGGAGTACATCTTGACAGTGTTTCATGAAAGAAATGGGTGGATAGCAGTGTACGATCAAAATGTTGAACAAACCTTGACCACTT
TGGTTTCAGTAAAACCGgcCCCCAGACACACACCTATTAAATTTGGTAAACCATATAGATACATGGTACATCGCATTGTAGGGAGAGAA
CACACTCTTGCATTGCGGTTGGCCGAGCTTGGAATGGTACCTAACGGTCAAAGCAACATCTGTACTGAGTGTTCAACTGATAGTACCCA
CTTCATGGATCATGTTCAGAAGAGGTGTTTATATACAATTAGGACAATGGGCTTGTTTGTTTGCACTAGACTCTACACTCCACGATGGA
TTGAAGCAGAACTGGCTCGCCAGAAGGTGCCTGCACATTACAAAAACGGAGCCTTTGGACCTATGTTGACGCATGAAGGTATCCCTCTC
ACACTTAGTCAAGCAGTGAGTGTTCTGTTGGGTAATAGTATCCGAGGCCCTCAAGGTGCAGGTACACTGATGCAGATATCCTATTATGG
AGTGGTAGCCCCGCCCGTGGTGCGCAAACACCGTACTAGGCGTCGGATGACCAGGCATGACAGCAGGACTTAATTATTGAATGTATTTT
TGTGCTTTGTTGTTTAATGTTTAATATAAATTAATGTTCAATATCCTCTATGTTAAAGGCACATTCGGCATAACAAGGTTTCTGGTTAA
ATTGTCGTTGTTCTGTTCCAAAAATGTGTGTCCTATGTTTCTTTCTCCTTTCTAGCATATAAAGTAGAATATGTATAACCCACTATCAA
ATTGCTTTGTTTGCTTAGATCATCGCGATAAATATCTTAATCATATTATCGCATTGGCGTGCGCCGTATGATCTGCCGCTTGAAGCCCG
GGCTGCACTGGACATGGACCCCCCAGGAGTGGCTCTAATGACTGAGAAATAAGAACAACGAT

>csrv1-S7 1534 nt
ATAAATTTCGACGGTAGATGACCATACCTTGGGCAAAGTACTAACCTGCTAACTCAGAATCCTCTATTTTTCTCTTAGTCTCGACATGG
CTGGCCAAACTGAGAACGGGAATGGTGGAAACCAATCTGGTCACAGGGAAGTGTTAAAGGCAGTAGATGGTAACTACCAACTTACACCT
CAAGTTGCATTGTCTAAGCCAGGTACTAAAGCAGTAGTGTCCACATTGTTGAATGAAGATAATGAATTGGTCAGAATGGATCAGTTGCA
GCGTTACCTGTCTAACAGCTACAGCAGTGGATTCTTACCCATGAATATGAATGCCAAACGCACTATCGCTGAAACCATCCTGGGCTATC
TGCCACCTGACGCGAAGATTAATGTTGAGGCTTTTCTCCGCTTGGTAATCGCCACCGAAAAGATTCGCAACGACGAGGCAATGGTCAGA

GTGGTGATTACAATGTGTGACGTGACAGGTAGGCATGCTAGGCAATTGCTCGTTAGGGACAAGTCAACCTATGAGTTCAGTGATTTAGA
AGAGGATTGGACGGCGAGTGGCGTTATGGTTGTAACATCAACAAAAAACAACTCTAAAGAGAGTAGTACCGTCATCATCGTGCCTGATT
CCCCAATTATGGGTGTAGTGTATGATAGGAATGATGAGACCATCTTGAACTTTTATGGATCATACGAGATTCGCAGGAAGGCCGGACTG
CAGTACGTCCTTCATGGGGTCAAGTCTGCTCCTTTTGGTTTGAGAGGCAAAGCTGCCACAATCGCCACCGCTGTGAATGAGGGACTGGC
TAACATTGTAACTAACTTTGGTTATGATGAACCTTCTACCTTTGTCCAAGCACCAAGAGACATACCAATTGTTGGTCAGATCAGTGGTA
CTGAAACCATTCCTGCATGTATCGCAGCTGGATTGAAGGGGTACAAAGAGCTTCTGATTGACGTTGATGTGTCCAGGGAAGATGATGAG
GGCCTACACACTTACACCGTTAAAGCGATCCAGCTTCCACAAGAGTTGGCCGCTGTTCTGATTGATGTAGATTCTTCTATGCCTTTAGG
GTACCTCCTCGCCTTGACTGAAGAACGATCAATGAATAGGGTCTCACTACTAGATGCAGTGAAATTTAAGGTCGACTTCGAAAAGAAAA
GGGCACAAGCAGCGGCTGAGTTTGTTAACTCAAACCCTGAGGAATTTGAAGACATTATGCAGAAGAGGAAAGAACAACTTGAAAAGATG
AGGgCTAGaCGCAGTGGAGCCACACGCTCGTCTGGCACTCAGAATCGTCCTACTCCTGTTCAAGCTGGCGCATCAATTAGATTCAACAT
GAAGAGGTAATGTGAGGGACAGGATGGATCGGAGTATCACGTAGGCATGGATGCGGGTTACGAGACCCTCAAGATCATCGAGGCTAGGA
AGGTGACTACGGGAGTTGTCTGCCCGCCTGATCTGCATAAGGTAGCAGAGGGAGGGAAGCCTTTCTGAATCGTGGTAAAGTATGGAAGT
CGTTCTGTCATGATCAACGAT

>csrv1-S8 1281 nt
ATAAATTCAGCCCAGGAAGAAGCTTGCATCGTCACACCTGTCCAGAGATTTCTAGTAATTTCTTCGAGAGTTGTGCATTGTTCGCATCA
TCATTCAAGATGTCCGATACTTCGAGCTATAAAACGAATGACCTTGGAGAAGACTTGTCTATTCCACCCAATCGTTCTGAAGCTCAAGA
AGGATCAGTTAGTCTACCCGCTTCTACGTTTGATGACATCATGAACAGACTAGCCTCACTAGAAGCACGTGCCGCTGAAACGGCCGCTG
AGAACCAAAAATTGCATCAGCAGATTGCACACATGAAAGGTGAAGATGAGCAAATAGTCACAAAGATGATCCAAATCACTGCAGGCATT
TATAGCTTTGACATTGAGGTACCTGAAGAGAAAGAGGATGAGTTAACTAAAGTACTACAGATATTACTGCGTGTTGGAGCAAATACACT
AAAACAGTATCCACATGGATTGACTGATGCCATGGAAACTGACAGCCATGTTATGATAGCCAATCAGGAGGCTCTGAGCTACTACGATA
AGCTACCCGTGCTGACTCGAGATGTTATTTGGGAGAAACCTGATTCTTTCACTCTCAGTGGATTCACTATTCATAGCGCCGCCGAACTC
ATGTCCGTCGTCGTTAAGGATGCAGGTGAAGATAACTACTCCTTTCTACATAATCTTGCTGGATATGAATGTGTGGTTACTGCTGGCGC
GCTGTTCACTAGAGTGCTGCTTTTGTCTAGATGGAGGAACACCTTCAATCTGGAGCAGAGAGCTATTCAGAAGATGATCCACTCTCCAC
AGTCCAGAAGGGAATCCAACAACAATGAGAGAGTAGGTAAGCGCTCGTTTGGTCGTGCCATTAGAGCTGAAGTCGCTCAATTGGATCAT
GAAGAGGAGCGTGTCTTATGCATTCCACGTCGTCCCGAAACGCCACCAATGTCTAGCCGCTTGTACTTCGACAATCCTGTGGAAGCTGA
CTTAGAGAATGCTTAAATGATCAGTACTCCTACTGACAAGATCAGCGATCTGTAGAATATTCTAATGTACTTCCTTTTTGTTTGACTTT
TCCCAAATTATTATTGTTTATTTACATATATGATGTGTGTTGATATATCTTATTAGATGTAGTAATTATATACTCAACATTTCTTAATT
ATGAATTAGTAATAGGTTGTTCTTATTCAGGACTACATAGACGATAATAGCCGACGCCTGAAATCTGCCTGAAGCTGGTGACGCGGCTA
GAGATGGTGGATGAGTCCTGAATAAGAACAACGAT

>csrv1-S9 1243 nt
ataaataTCTCTTGAGGCCTAGATTCGTAGACTGTGTTCACACTGATTGCGTTACGAAGAGTAAACTAGCAAACATGAAGAGCCAAACT
GGAAGTCGCGGTTTTTCTGCTAGATACGATTTGTGCCAACTCATCTGCGTGATACTAGCTACGGTGGGAGGAATGTCTCAGGTGATGTC
AATTATATTATCTGGATACGTACCTGCTGACTATCGTGAGACTTGCGAGTATGTTGGACATACACTTGCCATTCTGTGCTTTGCCATTC

AGATTGGACTTATGCGTTGGATGCGAAGTGACAAAGTTGCAATTGGCGTAGTGATGGATAAGATGTCTCGCTCCTTGTCAGCAGTCGGC
GTTGTTGGAGACCAAGATGTGATTAGCAAGCAAGGATCCAAATTGAATGATAGTGGGTATTTGAACTTGCTCGGTATGGCGCAGAGTCA
TTTTGATGGAATAGAACACCACCAACCTAAAGCTGAAGACACTGACTCTTTATTGGGTGATGAAAATGTTGAGTCTCTTGTTAAACTGA
ACGGTCAGTTTGTTATGATGATGAGCGGTACTGGCACAACTCTGCCTCTACGTTTGATGTCTGATTTCGTGAAAGCAGTGTTCACCACT
GAATTCAAGAATGACGACGCCACCTTCGTAGTGAAAGAAGACACCGTGCTACCAATGAGTTTGATGAACCATCCCATGACCGGTGCTGT
AGTGGAAAAGTATAACCCATTCTACATCTACGTAACATGGGGAGTGCGCGCTGATGGCACATGGTACGTTGCTAAAGGTACAGCCATTC
CTATGCCATCATATGTTAAAGATTGGGCTTTGAAAAGATCAGCAGCTGGTAGGCCACACTTCGCTTTCAAGAATGCTCCTTGGATTAAT
GACACCGTGAGCGTAACCACTGAACCCAAAACGAACAGAACTCCTGTAGTTATGGTTCCAAGTAGTGTATATAGTCTGTACCCTAACAT
CCACAAAGATGAGGTTATCGTTCCTGATGCCCCACCACCTTATCCTGTAGCTGCCAGCCCAGTGTAAACATTAACCAAGAAGCGCGCCT
ACCAGATCAACGATCTACCACATTGGTTTCCACACTAAATTATGTTTACCTCAATAAATGCGTGTATTCATTTAATTACAAATCTCGAT
TAGGCTCTTACGAACTCTTTCAGATGTGACTCGGCTACGTGGTAGGTCCCTGCATGGCGCCAGACGTGACAGATGAGGTAAACTAT

>csrv1-S10 1211 nt
ATTAAATTACAGTAACAGGTGAGCATGGCAGACAGCGTCTCTGATCGCTCGGGAGCTGGTCTCCCCCTGAGTTCTACTTTCGGCTATAA
GATTCTTTTGCAGAAGAAGGACTACGATCATGATGCCACTGTACCCGCAGATGCGATCTGGACACCTTTCGAGTCTAATGAAAAGTACC
AGAATGCAGATACGGATGCCTACCTCACGTTTTACCAAATAGGAACGAAGGTATTTCTGACCCTAACGAAAGCATCTCAAGATGTGGAT
CGTAGTTTACAACTTACTGTCACTTGGACCAGAAATGGAGGAGTAGGATTTACTGAAGACAGAGGCGGAATCATTAATTGGCTGCAACT
GTTATCTAAGACCAACTCAGTGGTGACGAAATCGCCGGTGATTTTAGTGTGTGATGATTTTCAAGGGTTATTTGTTCTGCTTCCTTGCA
ATTTGACTGAGGTGCACCAATGTGGCGACTTCGCTGTTAGAAAGAAGGTCCTGAAACCATTAGCTGTTCTAGCTCGTTTGTTGAACATT
GTACCATCTAGCAAAGAGGAGAACAACCTTTACATTCATAATATGCTAAAGGCACTTCATCTCAGAAGTGATGATTCCAGAGAATTGAT
TCCTTCGGATGGTGGAGCAAGAGTGTTAGCACCGTATCTAACCGATGAAGATACTGCATCTGTCATGCGTGGCGCACTTTTAGGATTGG
AACCAGGAGGCAAGGTGATTCGTTGGTGTAATCTACTCGTTGGGTATGATTTGGATCAGACCACGGCTGATTATTACTTCGACACCCTG
AAAATCGTGGGAGAAATGTGGAAGACTCCGAGTACGCCCCAGATAGATGTTGCATCCTGGGTCCCTCAATATAACGTGATGGTTCCTCT
ATTACCTACTCCGCCGACAGATCTTACATTGTATCAACAGTGGGTACATGATGCAGAAGAGACTGATCATAATGGCGCACTTGTACGAG
CTGGGAAGTATGGAATACAACAAGGATTGTTTACTGCAAATGAACCCTTCATTCCCCAATGGTTGAAGGCTCTACTTGATTCCTAAACG
TCACATTGAATGTTGGTCGTCCGATGATCTATTAACTATTTATTCATACATATTATCGCATTGGAGCGAGATATGCGACGAGTCTCTGT
GCTTTCCACTTAGTGTGTCCCTGCTAAGTGTGACTGTTACGATAGATCAACGAT

>csrv1-S11 1166 nt
ATAAACTACATAGGTGATTGACTTCTCCCACAACGCGATGAATTGGTCAAAATCATTAAACTTTCAGCCTTTTATGATGGACACTCGGC
CGCCCTTGAGTATAATCCCTGTGGTGAACCAGCTGGTCAAGATAGGCGAACAGGCTAATCAGAAATGGAGTATGGATGACAGAATTTAT
TTCGCCATCAGGAAGATAAATCCAGTATTCGTGACTAACAATTATGTAGCTTCGAAATCCGACTATACTATCCTGCAAATTCAGACCCA
ATTGATCGCGACGCTACCAGAAACACTTCTGTTCTTGGCATTCTCATACTATACTAGGGAGTATCAAGATAAGGTTGGTTTGATGAGAT
TCTACCCTGTTGCGGTGAAGAACATGATTCCAATCGTCACATATTTAAAGGACCGTGTTCACAATAACTTTGAGACGACTCTGGATCAG
GCGTATCGTATGAACGTCGTTAATTCGATGACTGCTTCAGAAGCGTTTGACTTGCTTGCAGGAATGTTGGCCACTACTAGGgCTGAGTT

GATTCAAAGAACGCGGATCTGTCCCGATCTCTTGAATGTGCTCAACAAGATGTCATTCTTGATAATCTATGGACCCAGTAGACCCACCA
TATTTTCATGGAAGCGACAAGGTTGAAGTGGATCGTAAATTGTTGGTATGCGAGGTACGCTTAAAATACTTGCTATATGGAACTGATAT
ATGGACATATATTAACCTGTGTTTACTGATTGTGCATTAGGATTAAGACGATTAGTTGCAATGGACCATAGTAGTAGTAGTCAACTGAT
GGTATCAATGATATGTAACTTTTGACTTAAGGGCAATATAATAATAATGAATATATCACATTACTTTGATTTCTCTTTGAAAAGCTTTG
GTGGAGTTGCGTTTTCATATATTTTACATCATCATTTTCCGTTGTTAATGTCCTTCTGCTTGTAGTGCTGTATGTATAGCTTGTTTGCT
GGGTTTGTCGGATAATATTACACCACTTTACATAATTACCGACCTGTATGTCGTAAGTGATTGATTGATGATCAGTAGATTTTGACGAT
CATCAACTTGTCGGAGCTCAACACCCACGACTGTAGTCTGCCGGAACTCAGTGACGAGGAGTAGCGACCCTTAGAGAGTGAAAGCAGTG
ATCAACGAT

>csrv1-S12 1130 nt
ATAAATTACGCGCTCGGACACGATAGTTACAGAGATCGTGGAAAGAGAACCAAGATTACTCTTGTTCTGTCTCTCTTACTTCTCTTTAC
TTTCTGTTGTGGTTTTGTCTGCACTTTTAGTTCTGTCATCAACTTCAGTTTTTTGTCAACTACTTCTTTTCTTTTCACCGCAAGAATGG
CGCAAAACATGAACTTAGATATCAACAATTTCGCACCCGTCATCTCTAGCATTGGTAGTCAGCTATGCTCATTGGCTGCGCATAAGTTG
CTCACCTCTCGCAAACAGTATGGTAATGGTGCCAAATCATTTGAAGAGTTTTATGCTGAAGTCGGAGGTATTATCGGTATGATGGGCAT
CAACTCTCAGACACCACCCGGCGTAAGAGAAGGGATCTTTAAGTTATATCAGAGTGCTTATTTATTTGGTGACCTATTCCCTGAAAACT
TTGGTGTACAGAATCAGCAACATATTAAATCACCCCCGGAGTTCACCGCTGTGGCAAGGAAATTAGAAGTCACTCGACCTCAGGGACAC
CGAGTTGACATAATTTTCAACCCACATGAAGTGCGATTCACTACGACGGGTGCAGTGCAGGCTGGAGATTTGCTTGGAACAGTCGCCCT
GCCAATTCAAGGCTCTGTGATTGCCACGCGCAACTGCAATGTGAACGCTATTGGAGGACAGCTTACTAGCACCAGACCGCAGATTACTG
CTTCGGCGCCTCTGCCTGCCCGGTCCGTGATTGTTGCCAGTTTTGATGCTACTGAAGTTGGATATGATGGTGGAGATCAATTATTTTCT
GTTGGAATCGCAATCCTTGCTAACAGATTCAACGGTTCAGTTGCCAACATGTCAAGACACAACTACATGATGCAAGTTTATGCTGCTAT
CCCAAATGGTATGTCAGACAGGGACTCATCAGCCATTATGCATTTTGCACAAGCAGCGCCTGTTGTTCTCGGTATGATGGAAAGACTTA
CCAGCGCTCCGAAGTGGGTTCTCGATTACTGATCTGACTGCGACTATCTAAGGCGTGCTGAGTAGGGAGGGACGCCGGTTTAAGGGTAG
TTTATCCAGGACTATCTTCCGGACCTAAAAGGCACGACGGTTCTATGGGCAAGATCAACGAT

S2. CLUSTAL multiple sequence alignment of segment 4 sequences. Conducted by MUSCLE (3.8)

```
S. serrata SZ-2007 HQ414130.1          --GAGAAACAGATGCGTAAAGAAAGGGAAAACGTGCAGATGCTGATGTTTCAACGTGGCA
E. sinensis WX-2012 KP638405.1         --GAGAAGCAGATCAACAAGGAGAAAGAGAATGTCAAGATGTTGATGTTTCAGCGCGGAA
C. sapidus S4                          CGGAGAAGCAAATCCGAAAAGAAAGAGAGAATGTTCAGATGCTGATGTTTCAGAGAGGGA
P virus                                CGGAGAAGCAAATCCGAAAAGAAAGAGAGAATGTTCAGATGCTGATGTTTCAGAGAGGGA
                                         ***** ** **    ** ** *  ** ** **  ***** **********  * ** *

S. serrata SZ-2007 HQ414130.1         TCCCTCAACATCCACACGAGTATTGTACCATTCCTACAGAAACTGATCATCTACTGTACA
E. sinensis WX-2012 KP638405.1        TTCCACAGAACCCTCACGAATACTGCACTATGGCATCGGAATCTGATCACCTATTGTACA
C. sapidus S4                         TACCTCAACAACCTCGCGAATATTGCACTCTGGCATCTGAATCGGACCACCTGTTATACA
P virus                               TACCTCAACAACCTGACGAATATTGTACTCTGGCATCTGAATCGGACCACCTGTTATACA
                                      * ** **  * **   *** ** ** **  *  *  * *** * ** ** **  * ****

S. serrata  SZ-2007 HQ414130.1        CCATTCATTCACTTGGAGCTCGAGCAGCTATATGTAGTATAGAGAACTTGTTATACTATC
E. sinensis WX-2012 KP638405.1        CCATTCATTCCCTTGGAGCGCGCGCTGCCATATGTAGTATTCAGAACCTGCTATACTATC
C. sapidus S4                         CCGTTCATTCCCTTGGAGCCAGGGCAGCTATTTGTAGCATACAGAACTTGTTGTACTATC
P virus                               CCGTTCATTCCCTTGGAGCCAGGGCAGCTATTTGTAGCATACAGAACTTGTTGTACTATC
                                      ** ******* ********  * ** ** ** ***** **  ***** ** * *******

S. serrata  SZ-2007 HQ414130.1        CCCTTCGCTCTGACGCATCGATTAGATTCAGAGGTCTGATAACCTATCT-GCGTACTCTT
E. sinensis WX-2012 KP638405.1        CAGTATCAAGGGATGCTCCTGTACGATTGAGAGGTTTAGTCACTTATCT-GGGAACACTT
C. sapidus S4                         CGTTGAGTGTCAATGCACCTTCCAGATTGAGAGGATTGGTGACATATTT-ACGGACTCTA
P virus                               CGTTGAGTGTCGATGCACCTTTCAGATTGAGAGGATTGGTGACATATTTAACGGACTCTA
                                      *  *        * **  *     **** *****  *  * ** *** *   * ** **

S. serrata  SZ-2007 HQ414130.1        GATTTTAGTAACCAGACAAATGTTATTGGCACTTTACACAGTTTAAAGCGTCATCCTCTA
E. sinensis WX-2012 KP638405.1        GACTTTGGCAATGAATCGAATGTGATCGGAACCCTGCAAAACTTGAAGAGACACCCCTTA
C. sapidus S4                         AATTTTAGCAATGAGACTAATGTGATAGGAACACTCCAGAACCTGAAGGATCATCCATTA
P virus                               AATTTTAGCAATGAGACTAATGTGATAGGAACACTCCAGAACCTGAAGGATCATCCATTA
                                       * *** * **  *  * ***** ** ** **  * ** *   * ***   ** **  **

S. serrata  SZ-2007 HQ414130.1        TTCAATGAATATTTAAGTCCAAGCTGGGATAAGATTAAAGCTTTCCTTATGATGATGAGT
E. sinensis WX-2012 KP638405.1        TTCAGTGAGTACATTAGTCCTGATTGGGATAAGATAAAGTCCTTCTTGATGATGATGAGC
C. sapidus S4                         TTCAATGAATATATCAGTCCTAGCTGGGACAAGATTAAAGCCTTCCTTATGATGATGAGT
P virus                               TTCAATGAATACATCAGTCCTCGCTGGGACAAGATTAAAGCCTTTCTTATGATGATGAGT
                                      **** *** **  * *****    ***** ***** **  * **  * ***********

S. serrata  SZ-2007 HQ414130.1        GCTACGGGAAAACCATTGTCTGTGTCAGATGATTATGAGGACTATATCACAGATGATAGG
E. sinensis WX-2012 KP638405.1        GCTACAGGTAACCCTCAATCCGTTTCTGATGATTACCAAGACTATATGACTGACGACAGA
C. sapidus S4                         GCGTCAGGGAAGCCCCAGTCTGTTTCAGATGATTATGAAGATTATTTGACTGATGACCGG
```

```
P virus                               GCGTCAGGGAAGCCCCAGTCTGTTTCAGATGATTATGAAGATTATTTGACTGATGACCGG
                                      **  * ** ** **    ** ** ** ********  * ** *** * ** ** **  *

S. serrata  SZ-2007 HQ414130.1        ATCACCACTGTGGTTTTCAAAGGTTTCACGGTCACACATTCAGCTTCGTCGAGGCGGTAT
E. sinensis WX-2012 KP638405.1        ATCACTACTGTAGTCTTCAAAGGATTTTCAGTTGTGCATGTAGTGTCGTCATGTAGGTAT
C. sapidus S4                         ATAACGACCGTGACTTTCAAAGGATTTACCGTAGTCCACGCCATCTCTTCGGTAAGGTAT
P virus                               ATAACGACCGTGACTTTCAAAGGATTTACCGTAGTCCACGCCATTTTTTCGGTAAGGTAT
                                      ** ** ** **    ******** **  * **    **       *  **     *****

S. serrata  SZ-2007 HQ414130.1        GGGCATAGTGAGATGGAAGGAGAGTTCTATACATGTGACGCCACTGCAGCCGATATGTTT
E. sinensis WX-2012 KP638405.1        GGTATGAGCGAGATGGAAGGAGAGTTCTATACCTGTAGTGCTACCGCCGCAGATATGTTC
C. sapidus S4                         GGTAACAGCGAGATGGAGGGAGAGTTCTACACAATTAGTGCGACCTCCGCTGATATGTTT
P virus                               GGTAACAGCGAGATGGAGGGAGAGTTTTACACAATTAGTGCGACCTCCGCTGATATGTTT
                                      **    ** ******** ******** ** **   *   ** **  * ** ********

S. serrata  SZ-2007 HQ414130.1        CGACATTTACTAGCCAATTATCAATATTCAGAAGATGTATGATAATGCGTGAGGAATGCA
E. sinensis WX-2012 KP638405.1        AGACACCTCATAGCAAACTACCAATACGAAGAAT----------GCGTGTGAGGAATGCG
C. sapidus S4                         AGACACTTGCTGGCCAACTACCACTATTCAGAGG----------GCGCGTGAGGGATGCA
P virus                               AGACACTTGCTGGCCAACTACCACTATTCAGAGG----------GCGCGTGAGGGATGCA
                                       ****  *  * ** ** ** ** **   ***              * ****** ****

S. serrata  SZ-2007 HQ414130.1        GGACGCCACACTCTCGGTTTGAACTGAATTACTGCTGGCGCAGGCACTGAGATCAACGAT
E. sinensis WX-2012 KP638405.1        AAACGCCACACTCTCGGTTTGAACTGAATTACTGCTGGCGCAGGCACTGAGATCAACGA-
C. sapidus S4                         GGACGCCACACTCTCGGTTTGAACTGAATTACTGCTGGCGCAGGCACTGAGATCAACGAT
P virus                               GGACGCCACACTTTCGGTTTGAACTGAATTACTGCTGGCGCAGGCACTGAGATCAACGAT
                                        ********** **********************************************
```

S3. Alignment file for inter-specific phylogenetic tree

```
#NEXUS
[written Fri Oct 02 11:00:41 EDT 2015 by Mesquite  version 3.02 (build 681) at Tsvetans-MacBook-Pro-
2.local/130.85.242.33]

BEGIN TAXA;
	TITLE Taxa;
	DIMENSIONS NTAX=16;
	TAXLABELS
		'MA_AR6_2013' 'GP2012_13' 'GP2012_4' 'GP2012_5' 'C0612_13' 'C0612_2' 'C0612_8' 'C0812_49' 'VA_X45'
'FL_A10' '0715_BR3_RDRP ' '0715_BR28_RDRP ' '0715_BR22_RDRP ' '0715_BR17_RDRP ' '0715_BR35_RDRP ' 'HM014010_MCRV '
	;

END;


BEGIN CHARACTERS;
	TITLE  Character_Matrix;
	DIMENSIONS  NCHAR=810;
	FORMAT DATATYPE = DNA GAP = - MISSING = ?;
	MATRIX
	'MA_AR6_2013'    ----------
TAAACAGCCTTACCACATAGGAGGATCCTTATTATTTCACAAGGTAGATGCTGTTGTGAATGCGAACAAGAAGTACCGTAGTGTTTCCAACAAGTATGGTCAGGGCATAAGTAATGCGA
TTCCACATATAGGTGTACCCGAAGTGATAGCAGTGTCTTCGGATGGACTGGCTGTTTGCTTGGCGTTAGATGTATCTGCCTTTGATGTCGCTCAGAAGTATACTGAGACAGAAATAGAA
CTTGCGATGCGTGATGGGTTCCTTGACTCTGAGACATCTATGGTTTCTGGAGAAACGGTGCTTGAGCGTATGAATCCAGCAGACTTGGCAAATAATCTACTCACAAACACACCTCCTAA
ATATAAATACCAAACGGCATTGGGTGATATCATCATTTTGCAACATGGTAATAGGTCAGGTGTACCCTGGACTGGAACTCAGAATGACTTAGTCAATGTGAGTAATCACCATATGGCTT
ACGATGAGTACAAAAAGCGTGTTGCGGAATTACAGCGACAAGGAAGAATCTCTGTCAATGTTAATGACAAGCATCACATCGTTCGTGTGTTTGGAGATGATTCTACATTCATCATGACG
TATGATGAGCCACCTACTGCTGACGAAGTACATTTGATGTGTGCCACTTTTGTTGAGAGTTACCAAGAAACTGCAGGTACACTAGGATTCGCCATCAACGCTAGGAAAGGAATGATAGG
TAGATACGGAAGTGAGTACCTCAAGAATACAGCTATCTATGGAAACATCAAATCAGTCAATCAGGTGAAGTTCCGTGGATCTGA--
	'GP2012_13'      ----------
TAAACAGCCTTACCACATAGGAGGATCCTTATTATTTCACAAGGTAGATGCTGTTGTGAATGCGAATAAGAAGTACCGTAGTGTTTCCAACAAGTATGGTCAGGGCATAAGTAATGCGA
TTCCACATATAGGTGTACCCGAAGTGATAGCAGTGTCTTCGGATGGACTGGCTGTTTGCTTGGCGTTAGATGTATCTGCCTTCGATGTCGCTCAGAAGTATACTGAGACAGAAATAGAA
CTTGCGATGCGTGATGGGTTCCTTGACTCTGAGACATCTATGGTTTCTGGAGAGACGGTGCTTGAGCGTATGAATCCAGCAGACTTGGCAAATAATCTACTCACAAACACACCTCCTAA
ATATAAATATCAAACGGCATTGGGTGATATCATCATTTTGCAACATGGTAATAGGTCAGGTGTACCCTGGACTGGAACTCAGAATGACTTAGTCAATGTGAGTAATCACCATATGGCTT
ACGATGAGTACAAAAAGCGTGTTGCGGAATTACAGCGACAAGGAAGAATCTCTGTCAATATTAATGACAAGCATCACATCGTTCGTGTGTTTGGAGATGATTCTACATTCATTATGACG
TATGATGAGCCACCTACTGCTGAGGAAGTACATTTGATGTGTGCCACTTTTGTTGAGAGTTACCAAGAAACTGCAGGTACACTAGGATTCGCCATCAACGCTAGGAAAGGAATGATAGG
TAGATACGGAAGTGAGTACCTCAAGAATACAGCTATCTATGGGAACATCAAATCAGTCAATCAGGTGAAGTTCCGTGGATCTGA--
	'GP2012_4'       ----------
TAAACAGCCTTACCACATAGGAGGATCCTTGTTATTTCACAAGGTAGATGCTGTTGTGAATGCGAACAAGAAGTACCGTAGTGTTTCCAACAAGTATGGTCAGGGCATAAGTAATGCGA
TTCCACATATAGGTGTACCCGAAGTGATAGCAGTGTCTTCGGATGGACTGGCTGTTTGCTTGGCGTTAGATGTATCTGCCTTTGATGTCGCTCAGAAGTATACTGAGACAGAAATAGAA
CTTGCGATGCGTGATGGGTTCCTTGACTCTGAGACATCTATGGTTTCTGGAGAAACGGTGCTTGAGCGTATGAATCCAGCAGACTTGGCAAATAATCTACTCACAAACACACCTCCTAA
ATATAAATACCAAACGGCATTGGGTGATATCATCATTTTGCAACATGGTAATAGGTCAGGTGTACCCTGGACTGGAACTCAGAATGACTTAGTCAATGTGAGTAATCACCATATGGCTT
ACGATGAGTACAAGAAGCGTGTTGCGGAATTACAGCGACAAGGAAGAATCTCTGTCAATGTTAATGACAAGCATCACATCGTTCGTGTGTTTGGAGATGATTCTACATTCATCATGACG
TATGATGAGCCACCTACTGCTGAGGAAGTACATTTGATGTGTGCCACTTTTGTTGAGAGTTACCAAGAAACTGCAGGTACACTAGGATTCGCCATCAACGCTAGGAAAGGAATGATAGG
TAGATACGGAAGTGAGTACCTCAAGAATACAGCTATCTATGGAAACATCAAATCAGTCAATCAGGTGAAGTTCCGTGGATCTGA--
```

```
        'GP2012_5'          ----------
TAAACAGCCTTACCACATAGGAGGATCCTTGTTATTTCACAAGGTAGATACTGTTGTGAATGCGAACAAGAAGTACCGTAGTGTTTCCAACAAGTATGGTCAGGGCATAAGTAATGCGA
TTCCACATATAGGTGTACCCGAAGTGATAGCAGTGTCTTCGGATGGACTGGCTGTTTGCTTGGCGTTAGATGTATCTGCCTTTGATGTCGCTCAGAAGTATACTGAGACAGAAATAGAA
CTTGCGATGCGTGATGGGTTCCTTGACTCTGAGACATCTATGGTTTCTGGAGAAACGGTGCTTGAGCGTATGAATCCAGCAGACTTGGCAAATAATCTACTCACAAACACACCTCCTAA
ATATAAATACCAAACGGCATTGGGTGATATCATCATTTTGCAACATGGTAATAGGTCAGGTGTACCCTGGACTGGAACTCAGAATGACTTAGTCAATGTGAGTAATCACCATATGGCTT
ACGATGAGTACAAAAAGCGTGTTGCGGAATTACAGCGACAAGGAAGAATCTCTGTCAATGTTAATGACAAGCATCACATCGTTCGTGTGTTTGGAGATGATTCTACATTCATCATGACG
TATGATGAGCCACCTACTGCTGAGGAAGTACATTTGATGTGTGCCACTTTTGTTGAGAGTTACCAAGAAACTGCAGGTACACTAGGATTCGCCATCAACGCTAGGAAAGGAATGATAGG
TAGATACGGAAGTGAGTACCTCAAGAATACAGCTATCTATGGAAACATCAAATCAGTCAATCAAGTGAAGTTCCGTGGATCTGA--
        'C0612_13'          ----------
TAAACAGCCTTACCACATAGGAGGATCCTTATTATTTCACAAGGTAGATGCTGTTGTGAATGCGAACAAGAAGTACCGTAGTGTTTCCAATAAGTATGGTCAGGGTATAAGTAATGCGA
TTCCACATATAGGTGTACCCGAAGTGATAGCAGTGTCTTCAGATGGACTGGCTGTTTGCTTGGCGTTAGATGTATCTGCCTTTGATGTCGCTCAGAAGTATACTGAGACAGAAATAGAA
CTTGCGATGCGTGATGGGTTCCTTGACTCTGAGACATCTATGGTTTCTGGAGAAACGGTGCTTGAGCGTATGAATCCAGCAGACTTGGCAAATAATCTACTCACAAACACACCTCCTAA
ATATAAATACCAAACGGCATTGGGTGATATCATCATTTTGCAACATGGTAATAGGTCAGGTGTACCCTGGACTGGAACTCAGAATGACTTAGTCAATGTGAGTAATCACCATATGGCTT
ACGATGAGTACAAAAAACGTGTTGCGGAATTACAGCGACAAGGAAGAATCTCTGTCAATGTTAATGACAAGCATCACATCGTTCGTGTGTTTGGAGATGATTCTACATTCATCATGACG
TATGATGAGCCACCTACTGCTGAGGAAGTACATTTAATGTGTGCCACTTTTGTTGAGAGTTACCAAGAAACTGCAGGTACACTAGGATTCGCCATCAACGCTAGGAAAGGAATGATAGG
TAGATACGGAAGTGAGTACCTCAAGAATACAGCTATCTATGGAAACATCAAATCAGTCAATCAGGTGAAGTTCCGTGGATCTGA--
        'C0612_2'           ----------
TAAACAGCCTTACCACATAGGAGGATCCTTATTATTTCACAAGGTAGATGCTGTTGTGAATGCGAACAAGAAGTACCGTAGTGTTTCCAACAAGTATGGTCAGGGCATAAGTAATGCGA
TTCCACATATAGGTGTACCCGAAGTGATAGCAGTGTCTTCGGATGGACTGGCTGTTTGCTTGGCGTTAGATGTATCTGCCTTTGATGTCGCTCAGAAGTATACTGAGACAGAAATAGAA
CTTGCGATGCGTGATGGGTTCCTTGACTCTGAGACATCTATGGTTTCTGGAGAAACGGTGCTTGAGCGTATGAATCCAGCAGACTTGGCAAATAATCTACTCACAAACACACCTCCTAA
ATATAAATACCAAACGGCATTGGGTGATATCATCATTTTGCAACATGGTAATAGGTCAGGTGTACCCTGGACTGGAACTCAGAATGACTTAGTCAATGTGAGTAATCACCATATGGCTT
ACGATGAGTACAAAAAGCGTGTTGCGGAATTACAGCGACAAGGAAGAATCTCTGTCAATGTTAATGACAAGCATCACATCGTTCGTGTGTTTGGAGATGATTCTACATTCATCATGACG
TATGATGAGCCACCTACTGCTGAGGAAGTACATTTGATGTGTGCCACTTTTGTTGAGAGTTACCAAGAAACTGCAGGTACACTAGGATTCGCCATCAACGCTAGGAAAGGAATGATAGG
TAGATACGGAAGTGAGTACCTCAAGAATACAGCTATCTATGGAAACATCAAATCAGTCAATCAGGTGAAGTTCCGTGGATCTGA--
        'C0612_8'           ----------
TAAACAGCCTTACCACATAGGAGGATCCTTATTATTTCACAAGGTAGATGCTGTTGTGAATGCGAACAAGAAGTACCGTAGTGTTTCCAACAAGTATGGTCAGGGCATAAGTAATGCGA
TTCCACATATAGGTGTACCCGAAGTGATAGCAGTGTCTTCGGATGGACTGGCTGTTTGTTTGGCGTTAGATGTATCTGCCTTTGATGTCGCTCAGAAGTATACTGAGACAGAAATAGAA
CTTGCGATGCGTGATGGGTTCCTTGACTCTGAGACATCTATGGTTTCTGGAGAAACGGTGCTTGAGCGTATGAATCCAGCAGACTTGGCAAATAATCTACTCACAAACACACCTCCTAA
ATATAAATACCAAACGGCATTGGGTGATATCATCATTTTGCAACATGGTAATAGGTCAGGTGTACCCTGGACTGGAACTCAGAATGACTTAGTCAATGTGAGTAATCACCATATGGCTT
ACGATGAGTACAAAAAGCGTGTTGCGGGATTACAGCGACAAGGAAGAATCTCTGTCAATGTTAATGACAAGCATCACATCGTTCGTGTGTTTGGAGATGATTCCACATTCATCATGACG
TATCATGAGCCACCTACTGCTGAGGAAGTACATTTGATGTGTGCCACTTTTGTTGAGAGTTACCAAGAAACTGCAGGTACACTAGGATTCGCCATCAACGCTAGGAAAGGAATGATAGG
TAGATACGGAAGTGAGTACCTCAAGAATACAGCTATCTATGGAAACATCAAATCAGTCAATCAGGTGAAGTTCCGTGGATCTGA--
        'C0812_49'          ----------
TAAACAGCCTTACCACATAGGAGGATCCTTATTATTTCACAAGGTAGATGCTGTTGTGAATGCGAACAAGAAGTACCGTAGTGTTTCCAACAAGTATGGTCAGGGCATAAGTAATGCGA
TTCCACATATAGGTGTACCCGAAGTGATAGCAGTGTCTTCGGATGGACTGGCTGTTTGCTTGGCGTTAGATGTATCTGCCTTTGATGTCGCTCAGAAGTATACTGAGACAGAAATAGAA
CTTGCGATGCGTGATGGGTTCCTTGACTCTGAGACATCTATGGTTTCTGGAGAAACGGTGCTTGAGCGTATGAATCCAGCAGACTTGGCAAATAATCTACTCACAAACACACCTCCTAA
ATATAAATACCAAACGGCATTGGGTGATATCATCATTTTGCAACATGGTAATAGGTCAGGCGTACCCTGGACTGGAACTCAGAATGACTTAGTCAATGTGAGTAATCACCATATGGCTT
ACGATGAGTACAAAAAGCGTGTTGCGGAATTACAGCGACAAGGAAGAATCTCTGTCAATGTTAATGACAAGCATCACATCGTTCGTGTGTTTGGAGATGATTCTACATTCATCATGACG
TATGATGAGCCACCTACTGCTGAGGAAGTACATTTGATGTGTGCCACTTTTGTTGAGAGTTACCAAGAAACTGCAGGTACACTAGGATTCGCCATCAACGCTAGGAAAGGAATGATAGG
TAGATACGGAAGTGAGTACCTCAAGAATACAGCTATCTATGGAAACATCAAATCAGTCAATCAGGTGAAGTTCCGTGGATCTGA--
        'VA_X45'            ----------
TAAACAGCCTTACCACATAGGAGGATCCTTGTTATTTCACAAGGTAGATGCTGTTGTGAATGCGAACAAGAAGTACCGTAGTGTTTCCAACAAGTATGGTCAGGGCATAAGTAATGCGA
TTCCACATATAGGTGTACCCGAAGTGATAGCAGTGTCTTCGGATGGACTGGCTGTTTGCTTGGCGTTAGATGTATCTGCCTTTGATGTCGCTCAGAAGTATACTGAGACAGAAATAGAA
CTTGCGATGCGTGATGGGTTCCTTGACTCTGAGACATCTATGGTTTCTGGAGAAACGGTGCTTGAGCGTATGAATCCAGCAGACTTGGCAAATAATCTACTCACAAACACACCTCCTAA
ATATAAATACCAAACGGCATTGGGTGATATCATTATTTTGCAACATGGTAATAGGTCAGGTGTACCCTGGACTGGAACTCAGAATGACTTAGTCAATGTGAGTAATCACCATATGGCTT
ACGATGAGTACAAAAAGCGTGTTGCGGAATTACAGCGACAAGGAAGAATCTCTGTCAATGTTAATGACAAGCATCACATCGTTCGTGTGTTTGGAGATGATTCTACATTCATCATGACG
```

```
TATGATGAGCCACCTACTCCTGAGGAAGTACATTTGATGTGTGCCACTTTTGTTGAGAGTTACCAAGAAACTGCAGGTACACTAGGATTCGCCATCAACGCTAGGAAAGGAATGATAGG
TAGATACGGAAGTGAGTACCTCAAGAATACAGCTATCTATGGAAACATCAAATCAGTCAATCAGGTGAAGTTCCGTGGATCTGA--
       'FL_A10'         ----------
TAAACAGCCTTACCACATAGGAGGATCCTTATTATTTCACAAGGTAGATGCTGTTGTCAATGCGAACAAGAAGTACCGTAGTGTTTCCAACAAGTATGGTCAGGGCATAAGTAATGCGA
TTCCACATATAGGTGTACCCGAAGTGATAGCAGTGTCTTCGGATGGACTGGCTGTTTGCTTGGCGTTAGATGTATCTGCCTTTGATGTCGCTCAGAAGTATACTGAGACAGAAATAGAA
CTTGCGATGCGTGATGGGTTCCTTGACTCTGAGACATCTATGGTTTCTGGAGAAACGGTGCTTGAGCGTATGAATCCAGCAGACTTGGCAAATAATCTGCTCACAAACACACCTCCTAG
ATATAAATATCAAACGGCATTGGGTGATATCATCATTTTGCAACATGGTAATAGGTCAGGTGTACCCTGGACTGGAACTCAGAATGACTTAGTCAATGTGAGTAATCACCATATGGCTT
ACGATGAGTACAAAAAGCGTGTTGCGGAATTACAGCGACAAGGAAGAATCTCTGTCAATGTTAATGACAAGCATCACATCGTTCGTGTGTTTGGAGATGATTCTACATTCATCATGACG
TATGATGAGCCACCTACTGCTGAAGAAGTACATTTGATGTGTGCCACTTTTGTTGAGAGTTACCAAGAAACTGCAGGTACACTAGGATTCGCCATCAACGCTAGGAAAGGAATGATAGG
TAGATACGGAAGTGAGTACCTCAAGAATACAGCTATCTATGGAAACATCAAATCAGTCAATCAGGTGAAGTTCCGTGGATCTGA--
       '0715_BR3_RDRP ' ----------
TAAACAGCCTTACCACATAGGAGGATCCTTATTATTTCACAAGGTAGACACTGTTGTGAATGCGAATAAGAAGTACCGTAGTGTTTCCAACAAGTATGGTCAGGGCATAAGTAATGCGA
TTCCACATATAGGTGTACCCGAAGTGATAGCAGTGTCTTCGGATGGACTGGCTGTTTGCTTGGCGTTAGATGTATCTGCCTTCGATGTCGCTCAGAAGTATACTGAGACAGAAATAGAA
CTTGCGATGCGTGATGGGTTCCTTGACTCTGAAACATCTATGATTTCTGGAGAGACGGTGCTTGAGCGTATGAATCCAGCAGACTTGGCAAATAATCTACTCACAAACACACCTCCTAA
ATATAAATACCAAACGGCATTGGGTGATATCATCATTTTGCAACATGGTAATAGGTCAGGTGTACCCTGGACTGGAACTCAGAATGACTTAGTCAATGTGAGTAATCACCATATGGCTT
ACGATGAGTACAAAAAGCGTGTTGCAGAATTACAGCGACAGGGAAGAATCTCTGTCAATGTTAATGACAAGCATCACATCGTTCGTGTGTTTGGAGATGATTCTACATTCATCATGACG
TATGATGAGCCACCTACTGCTGAGGAAGTACATTTGATGTGTGCCACTTTTGTTGAGAGTTACCAAGAAACTGCAGGTGCACTAGGATTCGCCATCAACGCTAGGAAAGGAATGATAGG
TAGATACGGAAGTGAGTACCTCAAGAATTCAGCTATCTATGGAAACATCAAATCAGTCAATCAGGTGAAGTTCCGTGGATCTGA--
       '0715_BR28_RDRP ' ----------
TAAACAGCCTTACCACATAGGAGGATCCTTATTATTTCACAAGGTAGACACTGTTGTGAATGCGAATAAGAAGTACCGTAGTGTTTCCAACAAGTATGGTCAGGGCATAAGTAATGCGA
TTCCACATATAGGTGTACCCGAAGTGATAGCAGTGTCTTCGGATGGACTGGCTGTTTGCTTGGCGTTAGATGTATCTGCCTTCGATGTCGCTCAGAAGTATACTGAGACAGAAATAGAA
CTTGCGATGCGTGATGGGTTCCTTGACTCTGAAACATCTATGATTTCTGGAGAGACGGTGCTTGAGCGTATGAATCCAGCAGACTTGGCAAATAATCTACTCACAAACACACCTCCTAA
ATATAAATACCAAACGGCATTGGGTGATATCATCATTTTGCAACATGGTAATAGGTCAGGTGTACCCTGGACTGGAACTCAGAATGACTTAGTCAATGTGAGTAATCACCATATGGCTT
ACGATGAGTACAAAAAGCGTGTTGCAGAATTACAGCGACAGGGAAGAATCTCTGTCAATGTTAATGACAAGCATCACATCGTTCGTGTGTTTGGAGATGATTCTACATTCATCATGACG
TATGATGAGCCACCTACTGCTGAGGAAGTACATTTGATGTGTGCCACTTTTGTTGAGAGTTACCAAGAAACTGCAGGTGCACTAGGATTCGCCATCAACGCTAGGAAAGGAATGATAGG
TAGATACGGAAGTGAGTACCTCAAGAATTCAGCTATCTATGGAAACATCAAATCAGTCAATCAGGTGAAGTTCCGTGGATCTGA--
       '0715_BR22_RDRP ' ----------
TAAACAGCCTTACCACATAGGAGGATCCTTATTATTTCACAAGGTAGACACTGTTGTGAATGCGAATAAGAAGTACCGTAGTGTTTCCAACAAGTATGGTCAGGGCATAAGTAATGCGA
TTCCACATATAGGTGTACCCGAAGTGATAGCAGTGTCTTCGGATGGACTGGCTGTTTGCTTGGCGTTAGATGTATCTGCCTTCGATGTCGCTCAGAAGTATACTGAGACAGAAATAGAA
CTTGCGATGCGTGATGGGTTCCTTGACTCTGAAACATCTATGATTTCTGGAGGGACGGTGCTTGAGCGTATGAATCCAGCAGACTTGGCAAATAATCTACTCACAAACACACCTCCTAA
ATATAAATACCAAACGGCATTGGGTGATATCATCATTTTGCAACATGGTAATAGGTCAGGTGTACCCTGGACTGGAACTCAGAATGACTTAGTCAATGTGAGTAATCACCATATGGCTT
ACGATGAGTACAAAAAGCGTGTTGCAGAATTACAGCGACAGGGAAGAATCTCTGTCAATGTTAATGACAAGCATCACATCGTTCGTGTGTTTGGAGATGATTCTACATTCATCATGACG
TATGATGAGCCACCTACTGCTGAGGAAGTACATTTGATGTGTGCCACTTTTGTTGAGAGTTACCAAGAAACTGCAGGTGCACTAGGATTCGCCATCAACGCTAGGAAAGGAATGATAGG
TAGATACGGAAGTGAGTACCTCAAGAATTCAGCTATCTATGGAAACATCAAATCAGTCATTCAGGTGAAGTTCCGTGGATCTGA--
       '0715_BR17_RDRP ' ----------
TAAACAGCCTTACCACATAGGAGGATCCTTATTATTTCACAAGGTAGACGCTGTTGTGAATGCGAATAAGAAGTACCGTAGTGTTTCCAACAAGTATGGTCAGGGCATAAGTAATGCGA
TTCCACATATAGGTGTACCCGAAGTGATAGCAGTGTCTTCGGATGGACTGGCTGTTTGCTTGGCGTTAGATGTATCTGCCTTCGATGTCGCTCAGAAGTATACTGAGACAGAAATAGAA
CTTGCGATGCGTGATGGGTTCCTTGACTTTGAGACATCTATGATTTCTGGAGAGACGGTGCTTGAGCGTATGAATCCAGCAGACTTGGCAAATAATCTACTCACAAACACACCTCCTAA
ATATAAATACCAAACGGCATTGGGTGATATCATCATTTTGCAACATGGTAATAGGTCAGGTGTACCCTGGACTGGAACTCAGAATGACTTAGTCAATGTGAGTAATCACCATATGGCTT
ACGATGAGTACAAAAAGCGTGTTGCAGAATTACAGCGACAGGGAAGAATCTCTGTCAATGTTAATGACAAGCATCACATCGTTCGTGTGTTTGGAGATGATTCTACATTCATCATGACG
TATGATGAGCCACCTACTGCTGAGGAAGTACATTTGATGTGTGCCACTTTTGTTGAGAGTTACCAAGAAACTGCAGGTGCGCTAGGATTCGCCATCAACGCTAGGAAAGGAATGATAGG
TAGATACGGAAGTGAGTACCTCAAGAATTCAGCTATCTATGGAAACATCAAATCAGTCAATCAGGTGAAGTTCCGTGGATCTGA--
       '0715_BR35_RDRP ' ----------
TAAACAGCCTTACCACATAGGAGGATCCTTATTATTTCACAAGGTAGACACTGTTGTGAATGCGAATAAGAAGTACCGTAGTGTTTCCAACAAGTATGGTCAGGGCATAAGTAATGCGA
TTCCACATATAGGTGTACCCGAAGTGATAGCAGTGTCTTCGGATGGACTGGCTGTTTGCTTGGCGTTAGATGTATCTGGCTTCGATGTCGCTCAGAAGTATACTGAGACAGAAATAGAA
CTTGCGATGCGTGATGGGTTCCTTGACTCTGAAACATCTATGATTTCTGGAGAGACGGTGCTTGAGCGTATGAATCCAGCAGACTTGGCAAATAATCTACTCACAAACACACCTCCTAA
ATATAAATACCAAACGGCATTGGGTGATATCATCATTTTGCAACATGGTAATAGGTCAGGTGTACCCTGGACTGGAACTCAGAATGACTTAGTCAATGTGAGTAATCACCATATGGCTT
```

```
ACGATGAGTACAAAAAGCGTGTTGCAGAATTACAGCGACAGGGAAGAATCTCTGTCAATGTTAATGACAAGCATCACATCGTTCGTGTGTTTGGAGATGATTCTACATTCATCATGACG
TATGATGAGCCACCTACTGCTGAGGAAGTACATTTGATGTGTGCCACTTTTGTTGAGAGTTCCCAAGAAACTGCAGGTTCACTAGGATTGTCCATCAACGCTAGGAAAGGAATGATAGG
TAGATACGGAAGTGAGTACCTCAAGAATTCAGCTATCTATGGAAACATCAAATCAGTCAATCAGGTGAAGTTCCGTGGATCTGA--
       'HM014010_MCRV ' ----------
TAAACAGCCTTATCACATTGCAGGATCTCTATTGTTCCATAAGGTGGACACTATTGTTAATGCCAACAAGAAATACCGTGGTGTGTCAAACAAATATGGTCAGGGTATTAGCAACGCCA
TTCCACATATTGGAGTTCCAGAGATCATTGCGGTATCATCGGACGGCATGGCAATTTGTCTGGCGTTAGATGTGTCTGCATTTGACGTGGCTCAAAAATATACTGAAGCTGATATAGAA
TTGGCTATGCGTGATGGCTTCCTTGATTCCGAAATTTCTATGATTTCTGGTGAAACCGTTTTGGAGCGCATGAATCCTGCGGATCTCGCTAACAACCTATTGACTAATACGCCACCTAG
GTACAAATACCAAACAGCACTTGGAGATATCATCATACTACAGCATGGCAACAGGTCTGGGGTTCCTTGGACGGGCACTCAAAATGACCTTGTCAATGTTAGTAATCATCATATGGCCT
ACGATGAGTACAAAAAACGTGTAGCTGAGTTACAACGTCAGGGCAAGATTTCCATCGATGTTAACGATAAACACCACATCGTTCGCGTCTTTGGAGATGATTCAACTTTCATCATGACT
TATGACGAACCACCTTCGGCTGAAGAGGTCCACCTTATGTGCGCAACGTTTGTGGAGAGCTACCAAGATACCGCGGGTACTCTAGGGTTTGCCATCAACGCTAGGAAGGGTATGATAGG
TAGGTACGGTAGTGAGTATCTCAAAAATTCTGCTATATATGGCAACATTAAGTCAGTTAATCAGGTTAAATTTCGTGGTTCTGA--

;


END;

BEGIN ASSUMPTIONS;
       TYPESET * UNTITLED   =  unord:  1 -  810;

END;

BEGIN NOTES;

       TAXABITS   TAXA = Taxa NAME = newlyAddedTaxon on =  1 -  16;

END;

BEGIN CODONS;
       CODONPOSSET * UNTITLED  =  N:  1- 8, 1: 9 - 810\3, 2: 10 - 808\3, 3: 11 - 809\3;

       CODESET * UNTITLED   =  universal:  1 -  810;


END;

BEGIN MESQUITECHARMODELS;
       ProbModelSet * UNTITLED   =  'Jukes-Cantor':  1 -  810;
END;

Begin MESQUITE;
              MESQUITESCRIPTVERSION 2;
              TITLE AUTO;
              tell ProjectCoordinator;
              timeSaved 1443798041455;
              getEmployee #mesquite.minimal.ManageTaxa.ManageTaxa;
              tell It;
```

```
			setID 0 1038481595233485206;
		endTell;
		getEmployee #mesquite.charMatrices.ManageCharacters.ManageCharacters;
		tell It;
			setID 0 7980344734932644583;
			mqVersion 302;
			checksumv 0 3 4278422652 null  getNumChars 810 numChars 810 getNumTaxa 16 numTaxa 16   short true
bits 15   states 15   sumSquaresStatesOnly 283482.0 sumSquares 283482.0 longCompressibleToShort false usingShortMatrix
true   NumFiles 1 NumMatrices 1;
			mqVersion;
		endTell;
		getWindow;
		tell It;
			suppress;
			setResourcesState false false 66;
			setPopoutState 400;
			setExplanationSize 0;
			setAnnotationSize 0;
			setFontIncAnnot 0;
			setFontIncExp 0;
			setSize 1916 720;
			setLocation 0 22;
			setFont SanSerif;
			setFontSize 10;
			getToolPalette;
			tell It;
			endTell;
			desuppress;
		endTell;
		getEmployee  #mesquite.trees.BasicTreeWindowCoord.BasicTreeWindowCoord;
		tell It;
			makeTreeWindow #1038481595233485206  #mesquite.trees.BasicTreeWindowMaker.BasicTreeWindowMaker;
			tell It;
				suppressEPCResponse;
				setTreeSource  #mesquite.trees.StoredTrees.StoredTrees;
				tell It;
					laxMode;
					setTreeBlock 1;
					toggleUseWeights off;
				endTell;
				setAssignedID 939.1443797775222.8673350073084462164;
				getTreeWindow;
				tell It;
					setExplanationSize 30;
					setAnnotationSize 20;
					setFontIncAnnot 0;
					setFontIncExp 0;
```

```
				setSize 1450 648;
				setLocation 0 22;
				setFont SanSerif;
				setFontSize 10;
				getToolPalette;
				tell It;
					setTool mesquite.trees.BasicTreeWindowMaker.BasicTreeWindow.collapse;
				endTell;
				getTreeDrawCoordinator
#mesquite.trees.BasicTreeDrawCoordinator.BasicTreeDrawCoordinator;
			tell It;
				suppress;
				setTreeDrawer  #mesquite.trees.SquareLineTree.SquareLineTree;
				tell It;
					setNodeLocs #mesquite.trees.NodeLocsStandard.NodeLocsStandard;
					tell It;
						branchLengthsToggle on;
						toggleScale on;
						toggleBroadScale off;
						toggleCenter on;
						toggleEven on;
						setFixedTaxonDistance 0;
					endTell;
					setEdgeWidth 4;
					showEdgeLines on;
					orientUp;
				endTell;
				setBackground White;
				setBranchColor Black;
				showNodeNumbers off;
				showBranchColors on;
				labelBranchLengths on;
				centerBrLenLabels on;
				showBrLensUnspecified on;
				showBrLenLabelsOnTerminals on;
				setBrLenLabelColor 0 0 255;
				setNumBrLenDecimals 6;
				desuppress;
				getEmployee #mesquite.trees.BasicDrawTaxonNames.BasicDrawTaxonNames;
				tell It;
					setColor Black;
					toggleColorPartition off;
					toggleColorAssigned on;
					toggleShadePartition off;
					toggleShowFootnotes on;
					toggleNodeLabels on;
					toggleCenterNodeNames off;
```

```
				toggleShowNames on;
				namesAngle ?;
			endTell;
		endTell;
			setTreeNumber 1;
			setTree '((1,10,9,8,7,6,2,3,4,5),(11,12,13,14,15),16);';
			setDrawingSizeMode 0;
			toggleLegendFloat on;
			scale 0;
			toggleTextOnTree off;
			showWindow;
			newAssistant  #mesquite.ancstates.TraceAllCharacters.TraceAllCharacters;
		tell It;
			toggleByCharacters on;
			toggleSelectedOnly on;
			toggleSelectedCharsOnly off;
			toggleShowTerminals off;
			getCharSource #mesquite.charMatrices.CharMatrixCoordIndep.CharMatrixCoordIndep;
			tell It;
				setCharacterSource #mesquite.charMatrices.StoredMatrices.StoredMatrices;
				tell It;
					setDataSet #7980344734932644583;
				endTell;
			endTell;
			getReconstructor #mesquite.ancstates.AncestralStatesAll.AncestralStatesAll;
			tell It;
				setMethod  #mesquite.parsimony.ParsAncestralStates.ParsAncestralStates;
				tell It;
					setModelSource  #mesquite.parsimony.CurrentParsModels.CurrentParsModels;
					toggleMPRsMode off;
				endTell;
			endTell;
			desuppress;
		endTell;
			newAssistant  #mesquite.trees.TreeLegendMaker.TreeLegendMaker;
		tell It;
			setOffsetsX 4;
			setOffsetsY 4;
			getLegendsVector;
			tell It;
			distributeCommands;
				setBounds 4 4 236 39;
				setOffsetX 4;
				setOffsetY 4;
			endDistributeCommands;
			endTell;
			newLegendItemNoCalc  #mesquite.trees.TreeValueUsingMatrix.TreeValueUsingMatrix;
```

```
				tell It;
					getEmployee #mesquite.parsimony.TreelengthForMatrix.TreelengthForMatrix;
					tell It;
						getEmployee #mesquite.parsimony.ParsCharSteps.ParsCharSteps;
						tell It;
							setModelSource
#mesquite.parsimony.CurrentParsModels.CurrentParsModels;
						endTell;
					endTell;
					getEmployee #mesquite.charMatrices.CharMatrixCoordIndep.CharMatrixCoordIndep;
					tell It;
						setCharacterSource #mesquite.charMatrices.StoredMatrices.StoredMatrices;
						tell It;
							setDataSet #7980344734932644583;
						endTell;
					endTell;
				endTell;
				calculate;
			endTell;
			endTell;
			desuppressEPCResponse;
			getEmployee #mesquite.trees.ColorBranches.ColorBranches;
			tell It;
				setColor Red;
				removeColor off;
			endTell;
			getEmployee #mesquite.ornamental.BranchNotes.BranchNotes;
			tell It;
				setAlwaysOn off;
			endTell;
			getEmployee #mesquite.ornamental.ColorTreeByPartition.ColorTreeByPartition;
			tell It;
				colorByPartition off;
			endTell;
			getEmployee #mesquite.ornamental.DrawTreeAssocDoubles.DrawTreeAssocDoubles;
			tell It;
				setOn on;
				toggleShow consensusFrequency;
				setDigits 4;
				writeAsPercentage off;
				toggleCentred off;
				toggleHorizontal on;
				toggleWhiteEdges on;
				setFontSize 10;
				setOffset 0  0;
			endTell;
			getEmployee #mesquite.ornamental.DrawTreeAssocStrings.DrawTreeAssocStrings;
```

```
                    tell It;
                          setOn on;
                          toggleCentred on;
                          toggleHorizontal on;
                          setFontSize 10;
                          setOffset 0  0;
                    endTell;
                    getEmployee #mesquite.trees.TreeInfoValues.TreeInfoValues;
                    tell It;
                          panelOpen false;
                    endTell;
              endTell;
        endTell;
        getEmployee  #mesquite.charMatrices.BasicDataWindowCoord.BasicDataWindowCoord;
        tell It;
              showDataWindow #7980344734932644583
#mesquite.charMatrices.BasicDataWindowMaker.BasicDataWindowMaker;
              tell It;
                    getWindow;
                    tell It;
                          setExplanationSize 30;
                          setAnnotationSize 20;
                          setFontIncAnnot 0;
                          setFontIncExp 0;
                          setSize 1450 648;
                          setLocation 0 22;
                          setFont SanSerif;
                          setFontSize 10;
                          getToolPalette;
                          tell It;
                                setTool blockMover;
                          endTell;
                          setActive;
                          setTool blockMover;
                          colorCells  #mesquite.charMatrices.ColorByState.ColorByState;
                    tell It;
                          setStateLimit 9;
                          toggleUniformMaximum on;
                    endTell;
                          colorRowNames  #mesquite.charMatrices.TaxonGroupColor.TaxonGroupColor;
                          colorColumnNames  #mesquite.charMatrices.CharGroupColor.CharGroupColor;
                          colorText  #mesquite.charMatrices.NoColor.NoColor;
                          setBackground White;
                          toggleShowNames on;
                          toggleShowTaxonNames on;
                          toggleTight off;
                          toggleThinRows off;
```

```
		toggleShowChanges off;
		toggleSeparateLines off;
		toggleShowStates on;
		toggleAutoWCharNames off;
		toggleAutoTaxonNames off;
		toggleShowDefaultCharNames off;
		toggleConstrainCW on;
		toggleBirdsEye off;
		toggleShowPaleGrid off;
		toggleShowPaleCellColors off;
		toggleShowPaleExcluded off;
		togglePaleInapplicable on;
		toggleShowBoldCellText off;
		toggleAllowAutosize off;
		toggleColorsPanel off;
		toggleLinkedScrolling on;
		toggleScrollLinkedTables off;
	endTell;
	showWindow;
	getWindow;
	tell It;
		forceAutosize;
	endTell;
	getEmployee #mesquite.charMatrices.ColorCells.ColorCells;
	tell It;
		setColor Red;
		removeColor off;
	endTell;
	getEmployee #mesquite.categ.StateNamesStrip.StateNamesStrip;
	tell It;
		showStrip off;
	endTell;
	getEmployee #mesquite.charMatrices.AnnotPanel.AnnotPanel;
	tell It;
		togglePanel off;
	endTell;
	getEmployee #mesquite.charMatrices.CharReferenceStrip.CharReferenceStrip;
	tell It;
		showStrip off;
	endTell;
	getEmployee #mesquite.charMatrices.QuickKeySelector.QuickKeySelector;
	tell It;
		autotabOff;
	endTell;
	getEmployee #mesquite.charMatrices.SelSummaryStrip.SelSummaryStrip;
	tell It;
		showStrip off;
```

```
					endTell;
					getEmployee #mesquite.align.AlignToDropped.AlignToDropped;
					tell It;
						gapCosts 8 3 2 2;
						subCosts  10 5 10 10 10 5 5 10 10 10 5 10;
					endTell;
					getEmployee #mesquite.molec.ColorByAA.ColorByAA;
					tell It;
						emphasizeDegeneracy off;
					endTell;
					getEmployee #mesquite.molec.SequenceInfoEditor.SequenceInfoEditor;
					tell It;
						panelOpen false;
					endTell;
				endTell;
			endTell;
			getEmployee  #mesquite.charMatrices.ManageCharacters.ManageCharacters;
			tell It;
				showCharacters #7980344734932644583 #mesquite.lists.CharacterList.CharacterList;
				tell It;
					setData 0;
					getWindow;
					tell It;
						newAssistant  #mesquite.lists.CharListInclusion.CharListInclusion;
						newAssistant  #mesquite.lists.CharListPartition.CharListPartition;
						newAssistant  #mesquite.lists.CharListCodonPos.CharListCodonPos;
						setExplanationSize 30;
						setAnnotationSize 20;
						setFontIncAnnot 0;
						setFontIncExp 0;
						setSize 1450 648;
						setLocation 0 22;
						setFont SanSerif;
						setFontSize 10;
						getToolPalette;
						tell It;
						endTell;
					endTell;
					showWindow;
					getEmployee #mesquite.lists.CharListAnnotPanel.CharListAnnotPanel;
					tell It;
						togglePanel off;
					endTell;
				endTell;
			endTell;
			endTell;
end;
```

S4. FASTA files for nucleotide pariwise comparisons between CsRV1 from different geographic regions.

```
>MA_AR6_2013
AAACAGCCTTACCACATAGGAGGATCCTTATTATTTCACAAGGTAGATG
CTGTTGTGAATGCGAACAAGAAGTACCGTAGTGTTTCCAACAAGTATGGT
CAGGGCATAAGTAATGCGATTCCACATATAGGTGTACCCGAAGTGATAGC
AGTGTCTTCGGATGGACTGGCTGTTTGCTTGGCGTTAGATGTATCTGCCT
TTGATGTCGCTCAGAAGTATACTGAGACAGAAATAGAACTTGCGATGCGT
GATGGGTTCCTTGACTCTGAGACATCTATGGTTTCTGGAGAAACGGTGCT
TGAGCGTATGAATCCAGCAGACTTGGCAAATAATCTACTCACAAACACAC
CTCCTAAATATAAATACCAAACGGCATTGGGTGATATCATCATTTTGCAA
CATGGTAATAGGTCAGGTGTACCCTGGACTGGAACTCAGAATGACTTAGT
CAATGTGAGTAATCACCATATGGCTTACGATGAGTACAAAAAGCGTGTTG
CGGAATTACAGCGACAAGGAAGAATCTCTGTCAATGTTAATGACAAGCAT
CACATCGTTCGTGTGTTTGGAGATGATTCTACATTCATCATGACGTATGA
TGAGCCACCTACTGCTGACGAAGTACATTTGATGTGTGCCACTTTTGTTG
AGAGTTACCAAGAAACTGCAGGTACACTAGGATTCGCCATCAACGCTAGG
AAAGGAATGATAGGTAGATACGGAAGTGAGTACCTCAAGAATACAGCTAT
CTATGGAAACATCAAATCAGTCAATCAGGTGAAGTTCCGTGGATCTGA

>GP2012_13
AAACAGCCTTACCACATAGGAGGATCCTTATTATTTCACAAGGTAGATG
CTGTTGTGAATGCGAATAAGAAGTACCGTAGTGTTTCCAACAAGTATGGT
CAGGGCATAAGTAATGCGATTCCACATATAGGTGTACCCGAAGTGATAGC
AGTGTCTTCGGATGGACTGGCTGTTTGCTTGGCGTTAGATGTATCTGCCT
TCGATGTCGCTCAGAAGTATACTGAGACAGAAATAGAACTTGCGATGCGT
GATGGGTTCCTTGACTCTGAGACATCTATGGTTTCTGGAGAGACGGTGCT
TGAGCGTATGAATCCAGCAGACTTGGCAAATAATCTACTCACAAACACAC
CTCCTAAATATAAATATCAAACGGCATTGGGTGATATCATCATTTTGCAA
CATGGTAATAGGTCAGGTGTACCCTGGACTGGAACTCAGAATGACTTAGT
CAATGTGAGTAATCACCATATGGCTTACGATGAGTACAAAAAGCGTGTTG
CGGAATTACAGCGACAAGGAAGAATCTCTGTCAATATTAATGACAAGCAT
CACATCGTTCGTGTGTTTGGAGATGATTCTACATTCATTATGACGTATGA
```

TGAGCCACCTACTGCTGAGGAAGTACATTTGATGTGTGCCACTTTTGTTG
AGAGTTACCAAGAAACTGCAGGTACACTAGGATTCGCCATCAACGCTAGG
AAAGGAATGATAGGTAGATACGGAAGTGAGTACCTCAAGAATACAGCTAT
CTATGGGAACATCAAATCAGTCAATCAGGTGAAGTTCCGTGGATCTGA

>GP2012_4
AAACAGCCTTACCACATAGGAGGATCCTTGTTATTTCACAAGGTAGATG
CTGTTGTGAATGCGAACAAGAAGTACCGTAGTGTTTCCAACAAGTATGGT
CAGGGCATAAGTAATGCGATTCCACATATAGGTGTACCCGAAGTGATAGC
AGTGTCTTCGGATGGACTGGCTGTTTGCTTGGCGTTAGATGTATCTGCCT
TTGATGTCGCTCAGAAGTATACTGAGACAGAAATAGAACTTGCGATGCGT
GATGGGTTCCTTGACTCTGAGACATCTATGGTTTCTGGAGAAACGGTGCT
TGAGCGTATGAATCCAGCAGACTTGGCAAATAATCTACTCACAAACACAC
CTCCTAAATATAAATACCAAACGGCATTGGGTGATATCATCATTTTGCAA
CATGGTAATAGGTCAGGTGTACCCTGGACTGGAACTCAGAATGACTTAGT
CAATGTGAGTAATCACCATATGGCTTACGATGAGTACAAGAAGCGTGTTG
CGGAATTACAGCGACAAGGAAGAATCTCTGTCAATGTTAATGACAAGCAT
CACATCGTTCGTGTGTTTGGAGATGATTCTACATTCATCATGACGTATGA
TGAGCCACCTACTGCTGAGGAAGTACATTTGATGTGTGCCACTTTTGTTG
AGAGTTACCAAGAAACTGCAGGTACACTAGGATTCGCCATCAACGCTAGG
AAAGGAATGATAGGTAGATACGGAAGTGAGTACCTCAAGAATACAGCTAT
CTATGGAAACATCAAATCAGTCAATCAGGTGAAGTTCCGTGGATCTGA

>GP2012_5
AAACAGCCTTACCACATAGGAGGATCCTTGTTATTTCACAAGGTAGATA
CTGTTGTGAATGCGAACAAGAAGTACCGTAGTGTTTCCAACAAGTATGGT
CAGGGCATAAGTAATGCGATTCCACATATAGGTGTACCCGAAGTGATAGC
AGTGTCTTCGGATGGACTGGCTGTTTGCTTGGCGTTAGATGTATCTGCCT
TTGATGTCGCTCAGAAGTATACTGAGACAGAAATAGAACTTGCGATGCGT
GATGGGTTCCTTGACTCTGAGACATCTATGGTTTCTGGAGAAACGGTGCT
TGAGCGTATGAATCCAGCAGACTTGGCAAATAATCTACTCACAAACACAC
CTCCTAAATATAAATACCAAACGGCATTGGGTGATATCATCATTTTGCAA
CATGGTAATAGGTCAGGTGTACCCTGGACTGGAACTCAGAATGACTTAGT
CAATGTGAGTAATCACCATATGGCTTACGATGAGTACAAAAAGCGTGTTG
CGGAATTACAGCGACAAGGAAGAATCTCTGTCAATGTTAATGACAAGCAT

CACATCGTTCGTGTGTTTGGAGATGATTCTACATTCATCATGACGTATGA
TGAGCCACCTACTGCTGAGGAAGTACATTTGATGTGTGCCACTTTTGTTG
AGAGTTACCAAGAAACTGCAGGTACACTAGGATTCGCCATCAACGCTAGG
AAAGGAATGATAGGTAGATACGGAAGTGAGTACCTCAAGAATACAGCTAT
CTATGGAAACATCAAATCAGTCAATCAAGTGAAGTTCCGTGGATCTGA

>C0612_13
AAACAGCCTTACCACATAGGAGGATCCTTATTATTTCACAAGGTAGATG
CTGTTGTGAATGCGAACAAGAAGTACCGTAGTGTTTCCAATAAGTATGGT
CAGGGTATAAGTAATGCGATTCCACATATAGGTGTACCCGAAGTGATAGC
AGTGTCTTCAGATGGACTGGCTGTTTGCTTGGCGTTAGATGTATCTGCCT
TTGATGTCGCTCAGAAGTATACTGAGACAGAAATAGAACTTGCGATGCGT
GATGGGTTCCTTGACTCTGAGACATCTATGGTTTCTGGAGAAACGGTGCT
TGAGCGTATGAATCCAGCAGACTTGGCAAATAATCTACTCACAAACACAC
CTCCTAAATATAAATACCAAACGGCATTGGGTGATATCATCATTTTGCAA
CATGGTAATAGGTCAGGTGTACCCTGGACTGGAACTCAGAATGACTTAGT
CAATGTGAGTAATCACCATATGGCTTACGATGAGTACAAAAAACGTGTTG
CGGAATTACAGCGACAAGGAAGAATCTCTGTCAATGTTAATGACAAGCAT
CACATCGTTCGTGTGTTTGGAGATGATTCTACATTCATCATGACGTATGA
TGAGCCACCTACTGCTGAGGAAGTACATTTAATGTGTGCCACTTTTGTTG
AGAGTTACCAAGAAACTGCAGGTACACTAGGATTCGCCATCAACGCTAGG
AAAGGAATGATAGGTAGATACGGAAGTGAGTACCTCAAGAATACAGCTAT
CTATGGAAACATCAAATCAGTCAATCAGGTGAAGTTCCGTGGATCTGA

>C0612_2
AAACAGCCTTACCACATAGGAGGATCCTTATTATTTCACAAGGTAGATG
CTGTTGTGAATGCGAACAAGAAGTACCGTAGTGTTTCCAACAAGTATGGT
CAGGGCATAAGTAATGCGATTCCACATATAGGTGTACCCGAAGTGATAGC
AGTGTCTTCGGATGGACTGGCTGTTTGCTTGGCGTTAGATGTATCTGCCT
TTGATGTCGCTCAGAAGTATACTGAGACAGAAATAGAACTTGCGATGCGT
GATGGGTTCCTTGACTCTGAGACATCTATGGTTTCTGGAGAAACGGTGCT
TGAGCGTATGAATCCAGCAGACTTGGCAAATAATCTACTCACAAACACAC
CTCCTAAATATAAATACCAAACGGCATTGGGTGATATCATCATTTTGCAA
CATGGTAATAGGTCAGGTGTACCCTGGACTGGAACTCAGAATGACTTAGT
CAATGTGAGTAATCACCATATGGCTTACGATGAGTACAAAAAGCGTGTTG

CGGAATTACAGCGACAAGGAAGAATCTCTGTCAATGTTAATGACAAGCAT
CACATCGTTCGTGTGTTTGGAGATGATTCTACATTCATCATGACGTATGA
TGAGCCACCTACTGCTGAGGAAGTACATTTGATGTGTGCCACTTTTGTTG
AGAGTTACCAAGAAACTGCAGGTACACTAGGATTCGCCATCAACGCTAGG
AAAGGAATGATAGGTAGATACGGAAGTGAGTACCTCAAGAATACAGCTAT
CTATGGAAACATCAAATCAGTCAATCAGGTGAAGTTCCGTGGATCTGA

>C0612_8
AAACAGCCTTACCACATAGGAGGATCCTTATTATTTCACAAGGTAGATG
CTGTTGTGAATGCGAACAAGAAGTACCGTAGTGTTTCCAACAAGTATGGT
CAGGGCATAAGTAATGCGATTCCACATATAGGTGTACCCGAAGTGATAGC
AGTGTCTTCGGATGGACTGGCTGTTTGTTTGGCGTTAGATGTATCTGCCT
TTGATGTCGCTCAGAAGTATACTGAGACAGAAATAGAACTTGCGATGCGT
GATGGGTTCCTTGACTCTGAGACATCTATGGTTTCTGGAGAAACGGTGCT
TGAGCGTATGAATCCAGCAGACTTGGCAAATAATCTACTCACAAACACAC
CTCCTAAATATAAATACCAAACGGCATTGGGTGATATCATCATTTTGCAA
CATGGTAATAGGTCAGGTGTACCCTGGACTGGAACTCAGAATGACTTAGT
CAATGTGAGTAATCACCATATGGCTTACGATGAGTACAAAAAGCGTGTTG
CGGGATTACAGCGACAAGGAAGAATCTCTGTCAATGTTAATGACAAGCAT
CACATCGTTCGTGTGTTTGGAGATGATTCCACATTCATCATGACGTATCA
TGAGCCACCTACTGCTGAGGAAGTACATTTGATGTGTGCCACTTTTGTTG
AGAGTTACCAAGAAACTGCAGGTACACTAGGATTCGCCATCAACGCTAGG
AAAGGAATGATAGGTAGATACGGAAGTGAGTACCTCAAGAATACAGCTAT
CTATGGAAACATCAAATCAGTCAATCAGGTGAAGTTCCGTGGATCTGA

>C0812_49
AAACAGCCTTACCACATAGGAGGATCCTTATTATTTCACAAGGTAGATG
CTGTTGTGAATGCGAACAAGAAGTACCGTAGTGTTTCCAACAAGTATGGT
CAGGGCATAAGTAATGCGATTCCACATATAGGTGTACCCGAAGTGATAGC
AGTGTCTTCGGATGGACTGGCTGTTTGCTTGGCGTTAGATGTATCTGCCT
TTGATGTCGCTCAGAAGTATACTGAGACAGAAATAGAACTTGCGATGCGT
GATGGGTTCCTTGACTCTGAGACATCTATGGTTTCTGGAGAAACGGTGCT
TGAGCGTATGAATCCAGCAGACTTGGCAAATAATCTACTCACAAACACAC
CTCCTAAATATAAATACCAAACGGCATTGGGTGATATCATCATTTTGCAA
CATGGTAATAGGTCAGGCGTACCCTGGACTGGAACTCAGAATGACTTAGT

CAATGTGAGTAATCACCATATGGCTTACGATGAGTACAAAAAGCGTGTTG
CGGAATTACAGCGACAAGGAAGAATCTCTGTCAATGTTAATGACAAGCAT
CACATCGTTCGTGTGTTTGGAGATGATTCTACATTCATCATGACGTATGA
TGAGCCACCTACTGCTGAGGAAGTACATTTGATGTGTGCCACTTTTGTTG
AGAGTTACCAAGAAACTGCAGGTACACTAGGATTCGCCATCAACGCTAGG
AAAGGAATGATAGGTAGATACGGAAGTGAGTACCTCAAGAATACAGCTAT
CTATGGAAACATCAAATCAGTCAATCAGGTGAAGTTCCGTGGATCTGA

>VA_X45
AAACAGCCTTACCACATAGGAGGATCCTTGTTATTTCACAAGGTAGATG
CTGTTGTGAATGCGAACAAGAAGTACCGTAGTGTTTCCAACAAGTATGGT
CAGGGCATAAGTAATGCGATTCCACATATAGGTGTACCCGAAGTGATAGC
AGTGTCTTCGGATGGACTGGCTGTTTGCTTGGCGTTAGATGTATCTGCCT
TTGATGTCGCTCAGAAGTATACTGAGACAGAAATAGAACTTGCGATGCGT
GATGGGTTCCTTGACTCTGAGACATCTATGGTTTCTGGAGAAACGGTGCT
TGAGCGTATGAATCCAGCAGACTTGGCAAATAATCTACTCACAAACACAC
CTCCTAAATATAAATACCAAACGGCATTGGGTGATATCATTATTTTGCAA
CATGGTAATAGGTCAGGTGTACCCTGGACTGGAACTCAGAATGACTTAGT
CAATGTGAGTAATCACCATATGGCTTACGATGAGTACAAAAAGCGTGTTG
CGGAATTACAGCGACAAGGAAGAATCTCTGTCAATGTTAATGACAAGCAT
CACATCGTTCGTGTGTTTGGAGATGATTCTACATTCATCATGACGTATGA
TGAGCCACCTACTCCTGAGGAAGTACATTTGATGTGTGCCACTTTTGTTG
AGAGTTACCAAGAAACTGCAGGTACACTAGGATTCGCCATCAACGCTAGG
AAAGGAATGATAGGTAGATACGGAAGTGAGTACCTCAAGAATACAGCTAT
CTATGGAAACATCAAATCAGTCAATCAGGTGAAGTTCCGTGGATCTGA

>FL_A10
AAACAGCCTTACCACATAGGAGGATCCTTATTATTTCACAAGGTAGATG
CTGTTGTCAATGCGAACAAGAAGTACCGTAGTGTTTCCAACAAGTATGGT
CAGGGCATAAGTAATGCGATTCCACATATAGGTGTACCCGAAGTGATAGC
AGTGTCTTCGGATGGACTGGCTGTTTGCTTGGCGTTAGATGTATCTGCCT
TTGATGTCGCTCAGAAGTATACTGAGACAGAAATAGAACTTGCGATGCGT
GATGGGTTCCTTGACTCTGAGACATCTATGGTTTCTGGAGAAACGGTGCT
TGAGCGTATGAATCCAGCAGACTTGGCAAATAATCTGCTCACAAACACAC
CTCCTAGATATAAATATCAAACGGCATTGGGTGATATCATCATTTTGCAA

CATGGTAATAGGTCAGGTGTACCCTGGACTGGAACTCAGAATGACTTAGT
CAATGTGAGTAATCACCATATGGCTTACGATGAGTACAAAAAGCGTGTTG
CGGAATTACAGCGACAAGGAAGAATCTCTGTCAATGTTAATGACAAGCAT
CACATCGTTCGTGTGTTTGGAGATGATTCTACATTCATCATGACGTATGA
TGAGCCACCTACTGCTGAAGAAGTACATTTGATGTGTGCCACTTTTGTTG
AGAGTTACCAAGAAACTGCAGGTACACTAGGATTCGCCATCAACGCTAGG
AAAGGAATGATAGGTAGATACGGAAGTGAGTACCTCAAGAATACAGCTAT
CTATGGAAACATCAAATCAGTCAATCAGGTGAAGTTCCGTGGATCTGA

>0715_BR3_RDRP
AAACAGCCTTACCACATAGGAGGATCCTTATTATTTCACAAGGTAGACA
CTGTTGTGAATGCGAATAAGAAGTACCGTAGTGTTTCCAACAAGTATGGT
CAGGGCATAAGTAATGCGATTCCACATATAGGTGTACCCGAAGTGATAGC
AGTGTCTTCGGATGGACTGGCTGTTTGCTTGGCGTTAGATGTATCTGCCT
TCGATGTCGCTCAGAAGTATACTGAGACAGAAATAGAACTTGCGATGCGT
GATGGGTTCCTTGACTCTGAAACATCTATGATTTCTGGAGAGACGGTGCT
TGAGCGTATGAATCCAGCAGACTTGGCAAATAATCTACTCACAAACACAC
CTCCTAAATATAAATACCAAACGGCATTGGGTGATATCATCATTTTGCAA
CATGGTAATAGGTCAGGTGTACCCTGGACTGGAACTCAGAATGACTTAGT
CAATGTGAGTAATCACCATATGGCTTACGATGAGTACAAAAAGCGTGTTG
CAGAATTACAGCGACAGGGAAGAATCTCTGTCAATGTTAATGACAAGCAT
CACATCGTTCGTGTGTTTGGAGATGATTCTACATTCATCATGACGTATGA
TGAGCCACCTACTGCTGAGGAAGTACATTTGATGTGTGCCACTTTTGTTG
AGAGTTACCAAGAAACTGCAGGTGCACTAGGATTCGCCATCAACGCTAGG
AAAGGAATGATAGGTAGATACGGAAGTGAGTACCTCAAGAATTCAGCTAT
CTATGGAAACATCAAATCAGTCAATCAGGTGAAGTTCCGTGGATCTGA

>0715_BR28_RDRP
AAACAGCCTTACCACATAGGAGGATCCTTATTATTTCACAAGGTAGACA
CTGTTGTGAATGCGAATAAGAAGTACCGTAGTGTTTCCAACAAGTATGGT
CAGGGCATAAGTAATGCGATTCCACATATAGGTGTACCCGAAGTGATAGC
AGTGTCTTCGGATGGACTGGCTGTTTGCTTGGCGTTAGATGTATCTGCCT
TCGATGTCGCTCAGAAGTATACTGAGACAGAAATAGAACTTGCGATGCGT
GATGGGTTCCTTGACTCTGAAACATCTATGATTTCTGGAGAGACGGTGCT
TGAGCGTATGAATCCAGCAGACTTGGCAAATAATCTACTCACAAACACAC

CTCCTAAATATAAATACCAAACGGCATTGGGTGATATCATCATTTTGCAA
CATGGTAATAGGTCAGGTGTACCCTGGACTGGAACTCAGAATGACTTAGT
CAATGTGAGTAATCACCATATGGCTTACGATGAGTACAAAAAGCGTGTTG
CAGAATTACAGCGACAGGGAAGAATCTCTGTCAATGTTAATGACAAGCAT
CACATCGTTCGTGTGTTTGGAGATGATTCTACATTCATCATGACGTATGA
TGAGCCACCTACTGCTGAGGAAGTACATTTGATGTGTGCCACTTTTGTTG
AGAGTTACCAAGAAACTGCAGGTGCACTAGGATTCGCCATCAACGCTAGG
AAAGGAATGATAGGTAGATACGGAAGTGAGTACCTCAAGAATTCAGCTAT
CTATGGAAACATCAAATCAGTCAATCAGGTGAAGTTCCGTGGATCTGA

>0715_BR22_RDRP
AAACAGCCTTACCACATAGGAGGATCCTTATTATTTCACAAGGTAGACA
CTGTTGTGAATGCGAATAAGAAGTACCGTAGTGTTTCCAACAAGTATGGT
CAGGGCATAAGTAATGCGATTCCACATATAGGTGTACCCGAAGTGATAGC
AGTGTCTTCGGATGGACTGGCTGTTTGCTTGGCGTTAGATGTATCTGCCT
TCGATGTCGCTCAGAAGTATACTGAGACAGAAATAGAACTTGCGATGCGT
GATGGGTTCCTTGACTCTGAAACATCTATGATTTCTGGAGGGACGGTGCT
TGAGCGTATGAATCCAGCAGACTTGGCAAATAATCTACTCACAAACACAC
CTCCTAAATATAAATACCAAACGGCATTGGGTGATATCATCATTTTGCAA
CATGGTAATAGGTCAGGTGTACCCTGGACTGGAACTCAGAATGACTTAGT
CAATGTGAGTAATCACCATATGGCTTACGATGAGTACAAAAAGCGTGTTG
CAGAATTACAGCGACAGGGAAGAATCTCTGTCAATGTTAATGACAAGCAT
CACATCGTTCGTGTGTTTGGAGATGATTCTACATTCATCATGACGTATGA
TGAGCCACCTACTGCTGAGGAAGTACATTTGATGTGTGCCACTTTTGTTG
AGAGTTACCAAGAAACTGCAGGTGCACTAGGATTCGCCATCAACGCTAGG
AAAGGAATGATAGGTAGATACGGAAGTGAGTACCTCAAGAATTCAGCTAT
CTATGGAAACATCAAATCAGTCATTCAGGTGAAGTTCCGTGGATCTGA

>0715_BR17_RDRP
AAACAGCCTTACCACATAGGAGGATCCTTATTATTTCACAAGGTAGACG
CTGTTGTGAATGCGAATAAGAAGTACCGTAGTGTTTCCAACAAGTATGGT
CAGGGCATAAGTAATGCGATTCCACATATAGGTGTACCCGAAGTGATAGC
AGTGTCTTCGGATGGACTGGCTGTTTGCTTGGCGTTAGATGTATCTGCCT
TCGATGTCGCTCAGAAGTATACTGAGACAGAAATAGAACTTGCGATGCGT
GATGGGTTCCTTGACTTTGAGACATCTATGATTTCTGGAGAGACGGTGCT

TGAGCGTATGAATCCAGCAGACTTGGCAAATAATCTACTCACAAACACAC
CTCCTAAATATAAATACCAAACGGCATTGGGTGATATCATCATTTTGCAA
CATGGTAATAGGTCAGGTGTACCCTGGACTGGAACTCAGAATGACTTAGT
CAATGTGAGTAATCACCATATGGCTTACGATGAGTACAAAAAGCGTGTTG
CAGAATTACAGCGACAGGGAAGAATCTCTGTCAATGTTAATGACAAGCAT
CACATCGTTCGTGTGTTTGGAGATGATTCTACATTCATCATGACGTATGA
TGAGCCACCTACTGCTGAGGAAGTACATTTGATGTGTGCCACTTTTGTTG
AGAGTTACCAAGAAACTGCAGGTGCGCTAGGATTCGCCATCAACGCTAGG
AAAGGAATGATAGGTAGATACGGAAGTGAGTACCTCAAGAATTCAGCTAT
CTATGGAAACATCAAATCAGTCAATCAGGTGAAGTTCCGTGGATCTGA

>0715_BR35_RDRP
AAACAGCCTTACCACATAGGAGGATCCTTATTATTTCACAAGGTAGACA
CTGTTGTGAATGCGAATAAGAAGTACCGTAGTGTTTCCAACAAGTATGGT
CAGGGCATAAGTAATGCGATTCCACATATAGGTGTACCCGAAGTGATAGC
AGTGTCTTCGGATGGACTGGCTGTTTGCTTGGCGTTAGATGTATCTGGCT
TCGATGTCGCTCAGAAGTATACTGAGACAGAAATAGAACTTGCGATGCGT
GATGGGTTCCTTGACTCTGAAACATCTATGATTTCTGGAGAGACGGTGCT
TGAGCGTATGAATCCAGCAGACTTGGCAAATAATCTACTCACAAACACAC
CTCCTAAATATAAATACCAAACGGCATTGGGTGATATCATCATTTTGCAA
CATGGTAATAGGTCAGGTGTACCCTGGACTGGAACTCAGAATGACTTAGT
CAATGTGAGTAATCACCATATGGCTTACGATGAGTACAAAAAGCGTGTTG
CAGAATTACAGCGACAGGGAAGAATCTCTGTCAATGTTAATGACAAGCAT
CACATCGTTCGTGTGTTTGGAGATGATTCTACATTCATCATGACGTATGA
TGAGCCACCTACTGCTGAGGAAGTACATTTGATGTGTGCCACTTTTGTTG
AGAGTTCCCAAGAAACTGCAGGTTCACTAGGATTGTCCATCAACGCTAGG
AAAGGAATGATAGGTAGATACGGAAGTGAGTACCTCAAGAATTCAGCTAT
CTATGGAAACATCAAATCAGTCAATCAGGTGAAGTTCCGTGGATCTGA

>HM014010_MCRV
AAACAGCCTTATCACATTGCAGGATCTCTATTGTTCCATAAGGTGGACA
CTATTGTTAATGCCAACAAGAAATACCGTGGTGTGTCAAACAAATATGGT
CAGGGTATTAGCAACGCCATTCCACATATTGGAGTTCCAGAGATCATTGC
GGTATCATCGGACGGCATGGCAATTTGTCTGGCGTTAGATGTGTCTGCAT
TTGACGTGGCTCAAAAATATACTGAAGCTGATATAGAATTGGCTATGCGT

GATGGCTTCCTTGATTCCGAAATTTCTATGATTTCTGGTGAAACCGTTTT
GGAGCGCATGAATCCTGCGGATCTCGCTAACAACCTATTGACTAATACGC
CACCTAGGTACAAATACCAAACAGCACTTGGAGATATCATCATACTACAG
CATGGCAACAGGTCTGGGGTTCCTTGGACGGGCACTCAAAATGACCTTGT
CAATGTTAGTAATCATCATATGGCCTACGATGAGTACAAAAAACGTGTAG
CTGAGTTACAACGTCAGGGCAAGATTTCCATCGATGTTAACGATAAACAC
CACATCGTTCGCGTCTTTGGAGATGATTCAACTTTCATCATGACTTATGA
CGAACCACCTTCGGCTGAAGAGGTCCACCTTATGTGCGCAACGTTTGTGG
AGAGCTACCAAGATACCGCGGGTACTCTAGGGTTTGCCATCAACGCTAGG
AAGGGTATGATAGGTAGGTACGGTAGTGAGTATCTCAAAAATTCTGCTAT
ATATGGCAACATTAAGTCAGTTAATCAGGTTAAATTTCGTGGTTCTGA

S5. Protein sequences used in alignment of geographic isolates.

>MA_AR6_2013
KQPYHIGGSLLFHKVDAVVNANKKYRSVSNKYGQGISNAIPHIGVPEVIAVSSDGLAVCLALDVSAFDVAQKYTETEIELAMRDGFLDS
ETSMVSGETVLERMNPADLANNLLTNTPPKYKYQTALGDIIILQHGNRSGVPWTGTQNDLVNVSNHHMAYDEYKKRVAELQRQGRISVN
VNDKHHIVRVFGDDSTFIMTYDEPPTADEVHLMCATFVESYQETAGTLGFAINARKGMIGRYGSEYLKNTAIYGNIKSVNQVKFRGS
>GP2012_13
KQPYHIGGSLLFHKVDAVVNANKKYRSVSNKYGQGISNAIPHIGVPEVIAVSSDGLAVCLALDVSAFDVAQKYTETEIELAMRDGFLDS
ETSMVSGETVLERMNPADLANNLLTNTPPKYKYQTALGDIIILQHGNRSGVPWTGTQNDLVNVSNHHMAYDEYKKRVAELQRQGRISVN
INDKHHIVRVFGDDSTFIMTYDEPPTAEEVHLMCATFVESYQETAGTLGFAINARKGMIGRYGSEYLKNTAIYGNIKSVNQVKFRGS
>GP2012_4
KQPYHIGGSLLFHKVDAVVNANKKYRSVSNKYGQGISNAIPHIGVPEVIAVSSDGLAVCLALDVSAFDVAQKYTETEIELAMRDGFLDS
ETSMVSGETVLERMNPADLANNLLTNTPPKYKYQTALGDIIILQHGNRSGVPWTGTQNDLVNVSNHHMAYDEYKKRVAELQRQGRISVN
VNDKHHIVRVFGDDSTFIMTYDEPPTAEEVHLMCATFVESYQETAGTLGFAINARKGMIGRYGSEYLKNTAIYGNIKSVNQVKFRGS
>GP2012_5
KQPYHIGGSLLFHKVDTVVNANKKYRSVSNKYGQGISNAIPHIGVPEVIAVSSDGLAVCLALDVSAFDVAQKYTETEIELAMRDGFLDS
ETSMVSGETVLERMNPADLANNLLTNTPPKYKYQTALGDIIILQHGNRSGVPWTGTQNDLVNVSNHHMAYDEYKKRVAELQRQGRISVN
VNDKHHIVRVFGDDSTFIMTYDEPPTAEEVHLMCATFVESYQETAGTLGFAINARKGMIGRYGSEYLKNTAIYGNIKSVNQVKFRGS
>C0612_13
KQPYHIGGSLLFHKVDAVVNANKKYRSVSNKYGQGISNAIPHIGVPEVIAVSSDGLAVCLALDVSAFDVAQKYTETEIELAMRDGFLDS
ETSMVSGETVLERMNPADLANNLLTNTPPKYKYQTALGDIIILQHGNRSGVPWTGTQNDLVNVSNHHMAYDEYKKRVAELQRQGRISVN
VNDKHHIVRVFGDDSTFIMTYDEPPTAEEVHLMCATFVESYQETAGTLGFAINARKGMIGRYGSEYLKNTAIYGNIKSVNQVKFRGS
>C0612_2

KQPYHIGGSLLFHKVDAVVNANKKYRSVSNKYGQGISNAIPHIGVPEVIAVSSDGLAVCLALDVSAFDVAQKYTETEIELAMRDGFLDS
ETSMVSGETVLERMNPADLANNLLTNTPPKYKYQTALGDIIILQHGNRSGVPWTGTQNDLVNVSNHHMAYDEYKKRVAELQRQGRISVN
VNDKHHIVRVFGDDSTFIMTYDEPPTAEEVHLMCATFVESYQETAGTLGFAINARKGMIGRYGSEYLKNTAIYGNIKSVNQVKFRGS
>C0612_8
KQPYHIGGSLLFHKVDAVVNANKKYRSVSNKYGQGISNAIPHIGVPEVIAVSSDGLAVCLALDVSAFDVAQKYTETEIELAMRDGFLDS
ETSMVSGETVLERMNPADLANNLLTNTPPKYKYQTALGDIIILQHGNRSGVPWTGTQNDLVNVSNHHMAYDEYKKRVAGLQRQGRISVN
VNDKHHIVRVFGDDSTFIMTYHEPPTAEEVHLMCATFVESYQETAGTLGFAINARKGMIGRYGSEYLKNTAIYGNIKSVNQVKFRGS
>C0812_49
KQPYHIGGSLLFHKVDAVVNANKKYRSVSNKYGQGISNAIPHIGVPEVIAVSSDGLAVCLALDVSAFDVAQKYTETEIELAMRDGFLDS
ETSMVSGETVLERMNPADLANNLLTNTPPKYKYQTALGDIIILQHGNRSGVPWTGTQNDLVNVSNHHMAYDEYKKRVAELQRQGRISVN
VNDKHHIVRVFGDDSTFIMTYDEPPTAEEVHLMCATFVESYQETAGTLGFAINARKGMIGRYGSEYLKNTAIYGNIKSVNQVKFRGS
>VA_X45
KQPYHIGGSLLFHKVDAVVNANKKYRSVSNKYGQGISNAIPHIGVPEVIAVSSDGLAVCLALDVSAFDVAQKYTETEIELAMRDGFLDS
ETSMVSGETVLERMNPADLANNLLTNTPPKYKYQTALGDIIILQHGNRSGVPWTGTQNDLVNVSNHHMAYDEYKKRVAELQRQGRISVN
VNDKHHIVRVFGDDSTFIMTYDEPPTPEEVHLMCATFVESYQETAGTLGFAINARKGMIGRYGSEYLKNTAIYGNIKSVNQVKFRGS
>FL_A10
KQPYHIGGSLLFHKVDAVVNANKKYRSVSNKYGQGISNAIPHIGVPEVIAVSSDGLAVCLALDVSAFDVAQKYTETEIELAMRDGFLDS
ETSMVSGETVLERMNPADLANNLLTNTPPRYKYQTALGDIIILQHGNRSGVPWTGTQNDLVNVSNHHMAYDEYKKRVAELQRQGRISVN
VNDKHHIVRVFGDDSTFIMTYDEPPTAEEVHLMCATFVESYQETAGTLGFAINARKGMIGRYGSEYLKNTAIYGNIKSVNQVKFRGS
>0715_BR3_RDRP
KQPYHIGGSLLFHKVDTVVNANKKYRSVSNKYGQGISNAIPHIGVPEVIAVSSDGLAVCLALDVSAFDVAQKYTETEIELAMRDGFLDS
ETSMISGETVLERMNPADLANNLLTNTPPKYKYQTALGDIIILQHGNRSGVPWTGTQNDLVNVSNHHMAYDEYKKRVAELQRQGRISVN
VNDKHHIVRVFGDDSTFIMTYDEPPTAEEVHLMCATFVESYQETAGALGFAINARKGMIGRYGSEYLKNSAIYGNIKSVNQVKFRGS
>0715_BR28_RDRP
KQPYHIGGSLLFHKVDTVVNANKKYRSVSNKYGQGISNAIPHIGVPEVIAVSSDGLAVCLALDVSAFDVAQKYTETEIELAMRDGFLDS
ETSMISGETVLERMNPADLANNLLTNTPPKYKYQTALGDIIILQHGNRSGVPWTGTQNDLVNVSNHHMAYDEYKKRVAELQRQGRISVN
VNDKHHIVRVFGDDSTFIMTYDEPPTAEEVHLMCATFVESYQETAGALGFAINARKGMIGRYGSEYLKNSAIYGNIKSVNQVKFRGS
>0715_BR22_RDRP
KQPYHIGGSLLFHKVDTVVNANKKYRSVSNKYGQGISNAIPHIGVPEVIAVSSDGLAVCLALDVSAFDVAQKYTETEIELAMRDGFLDS
ETSMISGGTVLERMNPADLANNLLTNTPPKYKYQTALGDIIILQHGNRSGVPWTGTQNDLVNVSNHHMAYDEYKKRVAELQRQGRISVN
VNDKHHIVRVFGDDSTFIMTYDEPPTAEEVHLMCATFVESYQETAGALGFAINARKGMIGRYGSEYLKNSAIYGNIKSVIQVKFRGS
>0715_BR17_RDRP
KQPYHIGGSLLFHKVDAVVNANKKYRSVSNKYGQGISNAIPHIGVPEVIAVSSDGLAVCLALDVSAFDVAQKYTETEIELAMRDGFLDF
ETSMISGETVLERMNPADLANNLLTNTPPKYKYQTALGDIIILQHGNRSGVPWTGTQNDLVNVSNHHMAYDEYKKRVAELQRQGRISVN
VNDKHHIVRVFGDDSTFIMTYDEPPTAEEVHLMCATFVESYQETAGALGFAINARKGMIGRYGSEYLKNSAIYGNIKSVNQVKFRGS

```
>0715_BR35_RDRP
KQPYHIGGSLLFHKVDTVVNANKKYRSVSNKYGQGISNAIPHIGVPEVIAVSSDGLAVCLALDVSGFDVAQKYTETEIELAMRDGFLDS
ETSMISGETVLERMNPADLANNLLTNTPPKYKYQTALGDIIILQHGNRSGVPWTGTQNDLVNVSNHHMAYDEYKKRVAELQRQGRISVN
VNDKHHIVRVFGDDSTFIMTYDEPPTAEEVHLMCATFVESSQETAGSLGLSINARKGMIGRYGSEYLKNSAIYGNIKSVNQVKFRGS
>HM014010_MCRV
KQPYHIAGSLLFHKVDTIVNANKKYRGVSNKYGQGISNAIPHIGVPEIIAVSSDGMAICLALDVSAFDVAQKYTEADIELAMRDGFLDS
EISMISGETVLERMNPADLANNLLTNTPPRYKYQTALGDIIILQHGNRSGVPWTGTQNDLVNVSNHHMAYDEYKKRVAELQRQGKISID
VNDKHHIVRVFGDDSTFIMTYDEPPSAEEVHLMCATFVESYQDTAGTLGFAINARKGMIGRYGSEYLKNSAIYGNIKSVNQVKFRGS
```

S6. Clustal Omega alignment of proteins. Amino acid differences are highlighted with red text.

```
CLUSTAL O(1.2.1) multiple sequence alignment
MA_AR6          KQPYHIGGSLLFHKVDAVVNANKKYRSVSNKYGQGISNAIPHIGVPEVIAVSSDGLAVCLALDVSAFDVAQKYTETEIELAMRDGFLDSE   90
NY_13           KQPYHIGGSLLFHKVDAVVNANKKYRSVSNKYGQGISNAIPHIGVPEVIAVSSDGLAVCLALDVSAFDVAQKYTETEIELAMRDGFLDSE   90
NY_4            KQPYHIGGSLLFHKVDAVVNANKKYRSVSNKYGQGISNAIPHIGVPEVIAVSSDGLAVCLALDVSAFDVAQKYTETEIELAMRDGFLDSE   90
NY_5            KQPYHIGGSLLFHKVDTVVNANKKYRSVSNKYGQGISNAIPHIGVPEVIAVSSDGLAVCLALDVSAFDVAQKYTETEIELAMRDGFLDSE   90
MD_13           KQPYHIGGSLLFHKVDAVVNANKKYRSVSNKYGQGISNAIPHIGVPEVIAVSSDGLAVCLALDVSAFDVAQKYTETEIELAMRDGFLDSE   90
MD_2            KQPYHIGGSLLFHKVDAVVNANKKYRSVSNKYGQGISNAIPHIGVPEVIAVSSDGLAVCLALDVSAFDVAQKYTETEIELAMRDGFLDSE   90
MD_8            KQPYHIGGSLLFHKVDAVVNANKKYRSVSNKYGQGISNAIPHIGVPEVIAVSSDGLAVCLALDVSAFDVAQKYTETEIELAMRDGFLDSE   90
MD_49           KQPYHIGGSLLFHKVDAVVNANKKYRSVSNKYGQGISNAIPHIGVPEVIAVSSDGLAVCLALDVSAFDVAQKYTETEIELAMRDGFLDSE   90
VA_X45          KQPYHIGGSLLFHKVDAVVNANKKYRSVSNKYGQGISNAIPHIGVPEVIAVSSDGLAVCLALDVSAFDVAQKYTETEIELAMRDGFLDSE   90
FL_A10          KQPYHIGGSLLFHKVDAVVNANKKYRSVSNKYGQGISNAIPHIGVPEVIAVSSDGLAVCLALDVSAFDVAQKYTETEIELAMRDGFLDSE   90
BR_3            KQPYHIGGSLLFHKVDTVVNANKKYRSVSNKYGQGISNAIPHIGVPEVIAVSSDGLAVCLALDVSAFDVAQKYTETEIELAMRDGFLDSE   90
BR_28           KQPYHIGGSLLFHKVDTVVNANKKYRSVSNKYGQGISNAIPHIGVPEVIAVSSDGLAVCLALDVSAFDVAQKYTETEIELAMRDGFLDSE   90
BR_22           KQPYHIGGSLLFHKVDTVVNANKKYRSVSNKYGQGISNAIPHIGVPEVIAVSSDGLAVCLALDVSAFDVAQKYTETEIELAMRDGFLDSE   90
BR_17           KQPYHIGGSLLFHKVDAVVNANKKYRSVSNKYGQGISNAIPHIGVPEVIAVSSDGLAVCLALDVSAFDVAQKYTETEIELAMRDGFLDFE   90
BR_35           KQPYHIGGSLLFHKVDTVVNANKKYRSVSNKYGQGISNAIPHIGVPEVIAVSSDGLAVCLALDVSGFDVAQKYTETEIELAMRDGFLDSE   90
                ****************:************************************************.********************** *

MA_AR6          TSMVSGETVLERMNPADLANNLLTNTPPKYKYQTALGDIIILQHGNRSGVPWTGTQNDLVNVSNHHMAYDEYKKRVAELQRQGRISVNVN   180
NY_13           TSMVSGETVLERMNPADLANNLLTNTPPKYKYQTALGDIIILQHGNRSGVPWTGTQNDLVNVSNHHMAYDEYKKRVAELQRQGRISVNIN   180
NY_4            TSMVSGETVLERMNPADLANNLLTNTPPKYKYQTALGDIIILQHGNRSGVPWTGTQNDLVNVSNHHMAYDEYKKRVAELQRQGRISVNVN   180
NY_5            TSMVSGETVLERMNPADLANNLLTNTPPKYKYQTALGDIIILQHGNRSGVPWTGTQNDLVNVSNHHMAYDEYKKRVAELQRQGRISVNVN   180
MD_13           TSMVSGETVLERMNPADLANNLLTNTPPKYKYQTALGDIIILQHGNRSGVPWTGTQNDLVNVSNHHMAYDEYKKRVAELQRQGRISVNVN   180
MD_2            TSMVSGETVLERMNPADLANNLLTNTPPKYKYQTALGDIIILQHGNRSGVPWTGTQNDLVNVSNHHMAYDEYKKRVAELQRQGRISVNVN   180
MD_8            TSMVSGETVLERMNPADLANNLLTNTPPKYKYQTALGDIIILQHGNRSGVPWTGTQNDLVNVSNHHMAYDEYKKRVAGLQRQGRISVNVN   180
MD_49           TSMVSGETVLERMNPADLANNLLTNTPPKYKYQTALGDIIILQHGNRSGVPWTGTQNDLVNVSNHHMAYDEYKKRVAELQRQGRISVNVN   180
VA_X45          TSMVSGETVLERMNPADLANNLLTNTPPKYKYQTALGDIIILQHGNRSGVPWTGTQNDLVNVSNHHMAYDEYKKRVAELQRQGRISVNVN   180
FL_A10          TSMVSGETVLERMNPADLANNLLTNTPPRYKYQTALGDIIILQHGNRSGVPWTGTQNDLVNVSNHHMAYDEYKKRVAELQRQGRISVNVN   180
BR_3            TSMISGETVLERMNPADLANNLLTNTPPKYKYQTALGDIIILQHGNRSGVPWTGTQNDLVNVSNHHMAYDEYKKRVAELQRQGRISVNVN   180
BR_28           TSMISGETVLERMNPADLANNLLTNTPPKYKYQTALGDIIILQHGNRSGVPWTGTQNDLVNVSNHHMAYDEYKKRVAELQRQGRISVNVN   180
BR_22           TSMISGGTVLERMNPADLANNLLTNTPPKYKYQTALGDIIILQHGNRSGVPWTGTQNDLVNVSNHHMAYDEYKKRVAELQRQGRISVNVN   180
BR_17           TSMISGETVLERMNPADLANNLLTNTPPKYKYQTALGDIIILQHGNRSGVPWTGTQNDLVNVSNHHMAYDEYKKRVAELQRQGRISVNVN   180
BR_35           TSMISGETVLERMNPADLANNLLTNTPPKYKYQTALGDIIILQHGNRSGVPWTGTQNDLVNVSNHHMAYDEYKKRVAELQRQGRISVNVN   180
                ***:** *********************:************************************************.**********:*

MA_AR6          DKHHIVRVFGDDSTFIMTYDEPPTADEVHLMCATFVESYQETAGTLGFAINARKGMIGRYGSEYLKNTAIYGNIKSVNQVKFRGS 265
NY_13           DKHHIVRVFGDDSTFIMTYDEPPTAEEVHLMCATFVESYQETAGTLGFAINARKGMIGRYGSEYLKNTAIYGNIKSVNQVKFRGS 265
NY_4            DKHHIVRVFGDDSTFIMTYDEPPTAEEVHLMCATFVESYQETAGTLGFAINARKGMIGRYGSEYLKNTAIYGNIKSVNQVKFRGS 265
NY_5            DKHHIVRVFGDDSTFIMTYDEPPTAEEVHLMCATFVESYQETAGTLGFAINARKGMIGRYGSEYLKNTAIYGNIKSVNQVKFRGS 265
MD_13           DKHHIVRVFGDDSTFIMTYDEPPTAEEVHLMCATFVESYQETAGTLGFAINARKGMIGRYGSEYLKNTAIYGNIKSVNQVKFRGS 265
MD_2            DKHHIVRVFGDDSTFIMTYDEPPTAEEVHLMCATFVESYQETAGTLGFAINARKGMIGRYGSEYLKNTAIYGNIKSVNQVKFRGS 265
MD_8            DKHHIVRVFGDDSTFIMTYHEPPTAEEVHLMCATFVESYQETAGTLGFAINARKGMIGRYGSEYLKNTAIYGNIKSVNQVKFRGS 265
MD_49           DKHHIVRVFGDDSTFIMTYDEPPTAEEVHLMCATFVESYQETAGTLGFAINARKGMIGRYGSEYLKNTAIYGNIKSVNQVKFRGS 265
```

```
VA_X45          DKHHIVRVFGDDSTFIMTYDEPPTPEEVHLMCATFVESYQETAGTLGFAINARKGMIGRYGSEYLKNTAIYGNIKSVNQVKFRGS 265
FL_A10          DKHHIVRVFGDDSTFIMTYDEPPTAEEVHLMCATFVESYQETAGTLGFAINARKGMIGRYGSEYLKNTAIYGNIKSVNQVKFRGS 265
BR_3            DKHHIVRVFGDDSTFIMTYDEPPTAEEVHLMCATFVESYQETAGALGFAINARKGMIGRYGSEYLKNSAIYGNIKSVNQVKFRGS 265
BR_28           DKHHIVRVFGDDSTFIMTYDEPPTAEEVHLMCATFVESYQETAGALGFAINARKGMIGRYGSEYLKNSAIYGNIKSVNQVKFRGS 265
BR_22           DKHHIVRVFGDDSTFIMTYDEPPTAEEVHLMCATFVESYQETAGALGFAINARKGMIGRYGSEYLKNSAIYGNIKSVIQVKFRGS 265
BR_17           DKHHIVRVFGDDSTFIMTYDEPPTAEEVHLMCATFVESYQETAGALGFAINARKGMIGRYGSEYLKNSAIYGNIKSVNQVKFRGS 265
BR_35           DKHHIVRVFGDDSTFIMTYDEPPTAEEVHLMCATFVESSQETAGSLGLSINARKGMIGRYGSEYLKNSAIYGNIKSVNQVKFRGS 265
                *******************.**** :************ *****:**::******************:********* *******
```
